# ATG Ubiquitination Is Required for Circumsporozoite Protein to Subvert Host Innate Immunity Against Rodent Malaria Liver Stage

**DOI:** 10.3389/fimmu.2022.815936

**Published:** 2022-02-09

**Authors:** Hong Zheng, Xiao Lu, Kai Li, Feng Zhu, Chenhao Zhao, Taiping Liu, Yan Ding, Yong Fu, Kun Zhang, Taoli Zhou, Jigang Dai, Yuzhang Wu, Wenyue Xu

**Affiliations:** ^1^Department of Pathogenic Biology, Army Medical University, Chongqing, China; ^2^The Institute of Immunology, Army Medical University, Chongqing, China; ^3^Department of Thoracic Surgery, Xinqiao Hospital, Army Medical University, Chongqing, China; ^4^Key Laboratory of Extreme Environmental Medicine, Ministry of Education of China, Chongqing, China

**Keywords:** *Plasmodium*, malaria, sporozoites, exo-erythrocytic forms, circumsporozoite protein, IFN-γ, ATGs, ubiquitination

## Abstract

Although exo-erythrocytic forms (EEFs) of liver stage malaria parasite in the parasitophorous vacuole (PV) are encountered with robust host innate immunity, EEFs can still survive and successfully complete the infection of hepatocytes, and the underlying mechanism is largely unknown. Here, we showed that sporozoite circumsporozoite protein (CSP) translocated from the parasitophorous vacuole into the hepatocyte cytoplasm significantly mediated the resistance to the killing of EEFs by interferon-gamma (IFN-γ). Attenuation of IFN-γ-mediated killing of EEFs by CSP was dependent on its ability to reduce the levels of autophagy-related genes (ATGs) in hepatocytes. The ATGs downregulation occurred through its enhanced ubiquitination mediated by E3 ligase NEDD4, an enzyme that was upregulated by CSP when it translocated from the cytoplasm into the nucleus of hepatocytes *via* its nuclear localization signal (NLS) domain. Thus, we have revealed an unrecognized role of CSP in subverting host innate immunity and shed new light for a prophylaxis strategy against liver-stage infection.

## Introduction

Malaria is still one of the most devastating diseases worldwide and is caused by infection with the genus *Plasmodium* that is initiated in the mammalian host by inoculation of sporozoites into the skin by Anopheles mosquitoes. From the skin, sporozoites travel to the liver *via* blood circulation, where they infect hepatocytes.

In the liver, sporozoites bind to highly sulfated heparin sulfate proteoglycans (HSPGs) on the hepatocyte surface by circumsporozoite protein (CSP), which triggers their invasion into hepatocytes (1). After invading hepatocytes, sporozoites inside a parasitophorous vacuole (PV) and transform into exo-erythrocytic forms (EEFs). To avoid fusion of the PV with lysosomes and to shield itself from the cytosol of the host, the PV membrane is modified by parasite-derived proteins, such as upregulated in infective sporozoite gene 4 (UIS4) and exported protein 1 (EXP-1) (2, 3). However, EEFs in the PV can be sensed by the Melanoma differentiation-associated gene 5 (MD-5) and elicit hepatocytes to generate a type-I IFN response (4). This recruits innate immune cells, such as natural killer (NK) and NK T cells, and eliminates the EEFs in the infected hepatocytes through the secretion of IFN-γ (5). The IFN-γ-mediated killing of EEFs was previously suggested to involve the production of nitric oxide (NO) (6, 7); it was recently reported to be regulated in an NO-independent manner (8). In addition, the infection of sporozoites could also induce *Plasmodium*-associated autophagy-related responses (PAAR) of hepatocytes to limit EEF development (9, 10). However, EEFs can still survive and successfully complete the infection of hepatocytes, and the underlying mechanism of parasite survival in this hostile environment remains largely unknown.

A previous study has shown that CSP, the major surface protein of sporozoites, translocating from the PV into the cytoplasm of hepatocytes, could promote liver stage development, which was thought to be associated with the reduced NF-κB activation in hepatocytes by CSP (11). However, in the present study we found that CSP enhanced the liver stage development through its resistance to the killing effect of IFN-γ on EEFs. Therefore, we uncovered a novel immune escape strategy for EEFs. Moreover, further study showed that the resistance mediated by CSP to the IFN-γ-killing of EEFs was dependent on its ability to downregulate autophagy-related genes (ATGs) through enhanced ubiquitination mediated by the E3 ligase NEDD4.

## Materials and Methods

### Parasites and Mice

*Plasmodium berghei* ANKA (*P.b* ANKA) was maintained in our laboratory, and the *P.b ANKA* CSP_mut_ parasite was constructed by mutation of pexel I-II of CSP using CRISPR-Cas9. All parasites were maintained by passage between Kunming mice (Swiss Webster strain) and *Anopheles stephensi*. ATG5^fl/fl^ mice (B6.129S-Atg5<tm1Myok>) were obtained from the RIKEN BioResource Center (Ibaraki, Japan), and Alb-Cre mice (B6.Cg-Speer6-ps1Tg(Alb-cre)21Mgn/J) were purchased from Jackson Laboratory (Bar Harbor, ME, USA). ATG5^fl/fl^ mice were crossed with *Alb-Cre* mice to conditionally delete the ATG5 cassette in hepatocytes. ATG5^fl/fl^ mice were used as controls. IFN-γR1 knockout mice were gifts from Dr. Bo Guo (Army Medical University, Chongqing, China). Kunming mice were purchased from the Experimental Animal Center of the Army Medical University (Chongqing, China). Animals were kept in a specific pathogen-free laboratory at the Institute of Immunology of Army Medical University. All methods were carried out in accordance with the approved Guide for the Care and Use of Laboratory Animals of the Army Medical University (approval number AMUWEC20171828).

### Mosquito Rearing and Infection

*Anopheles stephensi* (Hor strain) was maintained at 27°C and 70%–80% relative humidity and fed with a 5% sugar solution. For infection with the parasite, 3- to 5-day-old female adults were kept at 20–21°C and 70%–80% relative humidity and fed on *P.b* ANKA, *P.b* ANKA-RFP, or *P.b* ANKA-CSP_mut_-infected Kunming mice with gametocytemia ≥ 0.5%. Seven days after infection, the mosquitoes were dissected, and the oocysts on the midguts were examined under a light microscope.

### Cell Culture, Transfection, and Virus Infection

Both HepG2 and HEK293T cells were purchased from the American Type Culture Collection (Manassas, VA, USA). HepG2 cells were maintained in DMEM (Hyclone) and HEK293T cells were maintained in RPMI-1640 medium (Hyclone), supplemented with 10% FBS and 1% penicillin/streptomycin (Gibco) at 37°C in a humidified atmosphere with 5% CO_2_. For transfection, 3 × 10^4^ cells in 96-well, 1.2 × 10^5^ cells in 24-well plates, 1 × 10^6^ cells in 6-well plates, and 1 × 10^7^ cells in 10-cm dishes were transfected with 0.2, 0.8, 5, or 24 μg recombinant plasmid using 0.5, 2, 7.5 or 37.5 μL Lipofectamine 3000 (Invitrogen, CA, USA), respectively, according to the manufacturer’s protocol. For virus infection, 1 × 10^5^ HepG2 cells in 24-well plates were pretreated with 8 μg/mL polybrene (Santa Cruz, CA, USA) for 2 h, and then infected with Ad-mRFP-GRP-LC3 (Hanbio, Shanghai, China) at a multiplicity of infection (MOI) of 15. Four hours later, fresh cell culture containing 8 μg/mL polybrene was added. After 12 h of virus infection, the supernatant was discarded and replaced with fresh culture medium.

### Infection of *P. berghei* ANKA Sporozoites

At 19 days after infection with *P.b* ANKA, *P.b* ANKA-RFP, or *P.b* ANKA-CSP_mut_, infected female mosquitoes were extensively washed in sterile PBS, and the salivary glands were dissected and collected in RPMI 1640 medium containing 2.5 μg/mL amphotericin B (Sangon Biotech, Shanghai, China), 100 units/mL penicillin, and 100 μg/mL streptomycin (Beyotime, Beijing, China). The sporozoites were released from salivary glands and counted, and then incubated with HepG2 cells at a ratio of 1:3. Three hours after infection, the supernatant was discarded and replaced with fresh culture medium with the three antibiotics as described above. For the *in vivo* assay, ATG5^fl/fl^ and ATG5^fl/fl^-*Alb*-*Cre* were challenged with an intravenous injection of 1,000 sporozoites. Control and IFN-γ R1 knockout mice were challenged with an intravenous injection of 10,000 sporozoites.

### Construction of Recombinant Plasmids

The fragments of *P.b* ANKA full CSP (1020bp) were amplified from the genome of *P.b* ANKA using KOD-FX DNA polymerase (TOYOBO, Osaka, Japan). CSP-HA fragments were obtained by inserted HA-tag in C-terminal by PCR. CSPΔNLS (993 bp) and NLS (27 bp) fragments with initiation codons and HA-tag in C-terminal were synthesized by Sangon Biotech. CSP-HA and CSPΔNLS-HA with a 16-18 bp homologous sequence of the pcDNA3.1 vector at either the 5’ or 3’ end were obtained by PCR. The pcDNA3.1 vector was linearized using the restriction enzymes *HindIII* and *BamHI*. The fragments of CSP-HA and CSPΔNLS-HA were inserted into the pcDNA3.1 vector by seamless cloning according to the instructions of the In-fusion^®^ HD Cloning Kit (Clontech, Palo Alto, CA, USA). NLS-HA was inserted into pcDNA3.1 using the High-Efficient Ligation Reagent Ligation High (TOYOBO). The pcDNA3.1 vector was linearized using the restriction enzymes *BamHI* and *XholI* (TAKARA, Otsu, Japan) for ATG5 and LC3B, and with *KpnI* and *NotI* for ATG7. The pFLAG-cmv8 vector was linearized using the restriction enzymes *HindIII* and *EcoRI* for ATG5 and ATG7. Human LC3B, ATG5, and ATG7 were inserted into the pcDNA3.1 or pFLAG-cmv8 vector, respectively, by seamless cloning according to the instructions of the In-fusion^®^ HD Cloning Kit (Clontech). The pGL3-NEDD4 promoter reporter plasmid was constructed by cloning the NEDD4 promoter sequence (2000bp upstream form 1st exon) into the pGL3 vector through *KpnI*/*HindIII*. All recombinant plasmids were verified by DNA sequencing.

### Construction of CSP-Stably Transfected Cell Lines

The CSP coding sequence of *P.b* ANKA was cloned downstream of the CMV-7 promoter in the Lenti-pCDH plasmid (a gift from Professor Ying Wan, Army Medical University, China). HEK293T cells were transfected with Lenti-pCDH-CSP or Lenti-pCDH control plasmids with psPAX2 and pMD2.G packaging plasmids at a ratio of 20:15:5 using Lipofectamine 3000 (Invitrogen) to obtain the lentivirus. Seventy-two hours after transfection, the supernatant was filtered through a 0.45-μm filter (Millipore), and the lentivirus was collected by ultracentrifugation (20,000 rpm) at 4°C for 2 h. HepG2 cells were infected with Lenti-pCDH-CSP or Lenti-pCDH control lentivirus at an MOI of 15 for 48 h. Then, the positively infected cells were screened using 5 μg/mL puromycin (Gibco). CSP-stably transfected and control cells were cloned by a limited-dilution method in a 96-well plate, and 3 μg/mL puromycin was used to maintain the resistance of stably transfected cells. CSP expression was confirmed using immunofluorescence.

### Preparation of CSP Antibodies

The peptide containing three *P.b* ANKA CSP repeats, PAPPNANA-PAPPNANA- PAPPNANA, was synthesized by Wuhan GeneCreate Biological Engineering Co., Ltd. (Wuhan, China) and purified by high-performance liquid chromatography (HPLC) using a Waters XBridge C18 column (4.6 × 250 mm × 5 μm; Waters Corporation) on a Waters Corporation Prominence HPLC system (Massachusetts, USA).

The peptide was conjugated to bovine serum albumin (BSA). The resulting conjugated peptide was emulsified with Freund’s adjuvant (Sigma Aldrich) and immunized in a specific pathogen-free-grade rabbit at 0, 2, 4, and 6 weeks. Seven days after the final immunization, rabbit serum was collected, and the titer of the antibody against the epitope was detected using an enzyme-linked immunosorbent assay (cat. no. 44-2404-21, Nunc MaxiSorp flat bottom, Nalge Nunc International, Penfield, NY, USA). Protein G Sepharose (GE Healthcare) was used to purify the CSP antibody.

### Immunofluorescence Assay

HepG2 cells (1 × 10^5^) were placed on a 14-mm-diameter slide in a 24-well plate. To detect the distribution of CSP, HepG2 cells were infected with CSP_wt_ or CSP_mut_ sporozoites. Three hours after infection, the cells were washed with PBS to remove non-invaded sporozoites and incubated with fresh DMEM (Hyclone) containing 10% FBS (Gibco) and three antibiotics. Twenty-four hours later, the slides were immobilized with 4% paraformaldehyde (Sangon Biotech) for 10 min, permeabilized with 0.1% Triton-X 100 (Amersco, Albany, NY, USA) for 15 min, and then blocked with PBS containing 5% BSA and 0.02% Tween-20 for 1 h at room temperature. The cells were labeled with 1:500 goat anti-UIS4 (Sicgen, Cantanhede, Portugal) overnight at 4°C and 1:100 green donkey anti-goat secondary antibody (Abbkine, Wuhan, China) for 1 h at room temperature. After washing with PBS three times, the cells were labeled with 1:500 rabbit anti-CSP overnight at 4°C and 1:100 red donkey anti-rabbit secondary antibody (Abbkine) for 1 h at room temperature. Nuclei were counterstained with DAPI (Beyotime) for 5 min at room temperature.

For invasion capacity comparison, UIS4 staining was used to identify the CSP_wt_ and CSP_mut_ parasites in HepG2 cells, and images were obtained at 6 h after infection. To evaluate the co-localization of EEFs and LC3, control and CSP-stably transfected HepG2 cells were transfected with LC3B-RFP plasmid and infected with CSP_wt_ or CSP_mut_ sporozoites, and then labeled with goat anti-UIS4 and the secondary antibody as described above. To observe the effect of CSP on the IFN-γ-mediated killing of EEFs, control and CSP-stably transfected HepG2 cells were pre-treated with 1 U/mL recombinant human IFN-γ (Peprotech, Rocky Hill, NJ, USA) or equivoluminal medium for 6 h. The cells were then infected with CSP_wt_ or CSP_mut_ as stated above and co-treated with IFN-γ for 46 h.

For evaluating the effect of autophagy regulation on IFN-γ-mediated killing, HepG2 cells were pre-treated with 0.2 U/mL, 0.5 U/mL, and 1 U/mL IFN-γ for 6 h, and then infected with *P.b* ANKA-RFP sporozoites at a ratio of 1:3 and co-treated with IFN-γ and 0.25 μg/mL rapamycin, 10 μM LY294002 (Cell Signaling Technology, Danvers, MA, USA), 100nM wortmannin (Sigma-Aldrich), 1μM TAK-243 (MCE, NJ, USA) or equivoluminal medium for 24 or 46 h, respectively.

For autophagy flux observation, the control and CSP-stably transfected HepG2 cells were pre-treated with 8 μg/mL polybrene for 2 h, and then infected with adenovirus containing tandem GFP-RFP-LC3 (Hanbio, Shanghai, China). Since the cell culture was changed, the cells were treated with 0.25 μg/mL rapamycin (Cell Signaling Technology) or equivoluminal medium for 24 h.

For nuclear translocation of CSP observation, HepG2 cells were transfected with pcDNA3.1-CSP-HA and pcDNA3.1-CSPΔNLS-HA plasmids for 24 h. The cells were then labeled with rabbit anti-HA and the secondary antibody, as stated above.

The cells were immobilized, penetrated, and stained with DAPI. After washing with PBS, the slides were mounted on a microscope slide using DAKO Fluorescence Mounting Medium (Agilent). LSM 780 NLO microscope systems and ZEN Imaging Software (Zeiss) were used for image acquisition and export. Image J software (NIH) was used to analyze the number and size of the EEFs.

### Total RNA Extraction and Real-Time PCR

For parasite burden detection, the livers of mice were dissected at 46 h after infection with CSP_wt_ or CSP_mut_ sporozoites, and homogenized in 1.5 mL Trizol (Invitrogen), and HepG2 cells were collected 46 h after infection with CSP_wt_ or CSP_mut_ sporozoites and lysed with 1 mL Trizol (Invitrogen). Liver homogenate (100 μL) or 1 mL of cell lysis were used for total RNA extraction using TRIzol in accordance with the manufacturer’s instructions. cDNA was synthesized from equivalent total RNA using the PrimeScript™ RT reagent Kit with gDNA Eraser (TAKARA) in accordance with the manufacturer’s instructions. The parasite load in the livers and HepG2 cells was evaluated by Taqman PCR with primers and probes for 18S rRNA and GAPDH following the manufacturer’s instructions of Premix Ex Taq™ (Probe qPCR) (TAKARA). The mRNA levels of *IFN-γ*, *iNOS*, *LC3B*, *ATG3*, *ATG5*, *ATG7*, *STUB1*, *SYVN1*, *CBL*, *SMURF1*, *PAFAH1B1*, and *NEDD4* were evaluated using TB Green™ Premix Ex Taq™ II (Tli RNaseH Plus) (TAKARA). CFX96 Touch™ Real-Time PCR Detection System and CFX ManagerTM Software (Bio-Rad, Hercules, CA, USA) were used for RT-PCR data collection and analysis. The annealing temperature used for the above two RT-qPCR experiments was 60°C.

### Western Blot and Immunoprecipitation

To investigate the influence of CSP overexpression on the protein levels of ATGs, 1 × 10^6^ control and CSP-stably transfected HepG2 cells were incubated in 6-well plates and treated with 0.25 μg/mL rapamycin for 0, 6, 12, and 24 h. To observe the influence of CSP overexpression on the half-life of ATG proteins, CSP-stably transfected HepG2 and control cells were treated with 20 μM cycloheximide (CHX, Cell Signaling Technology) or 10 μM MG132 (Cell Signaling Technology) for 0, 6, 12, and 24 h. To test whether the degeneration of ATGs was dependent on the ubiquitin-proteasome system, CSP-stably transfected HepG2 and control cells were treated with IFN-γ, 1μM TAK-243 or both for 24 h. Uniformly, the cells were lysed with 300 μL RIPA lysis buffer containing a protease and phosphatase inhibitor cocktail (Thermo Fisher) at various time points and protein concentrations were detected using the BCA Protein Assay Kit (Sangon Biotech). Total proteins (20 μg equivalent) were then separated by 10% or 12% sodium dodecyl sulfate-polyacrylamide gel electrophoresis (SDS-PAGE; Bio-Rad), transferred using a 0.22-μm polyvinylidene fluoride (PVDF) filter (Millipore) and blocked with blocking solution (Sangon Biotech). The PVDF filter was incubated with 1:2000 rabbit anti-LC3B (Sigma-Aldrich), 1:2000 rabbit anti-ATG3 (Abcam, Cambridge, UK), 1:2000 rabbit anti-ATG5 (Invitrogen), 1:2000 rabbit anti-ATG7 (Sigma-Aldrich), 1:2000 rabbit anti-P62 (Sigma-Aldrich), 1:2000 rabbit anti-Beclin-1 (Cell Signaling Technology), and 1:2000 mouse anti-β-actin (Sigma-Aldrich) overnight at 4°C. Then, the PVDF filter was washed three times with TBS solution containing 1% Tween-20 (Amersco) and incubated with 1:20000 goat anti-rabbit or anti-mouse IgG-HRP secondary antibody (Santa Cruz) for 1 h at room temperature.

The ubiquitination of ATGs was detected by immunoprecipitation, as previously described (12). To detect the influence of CSP overexpression on the ubiquitination of ATGs, 2 × 10^6^ CSP-stably transfected HepG2 and control cells in two 6-wells were transfected with 2μg His-UB, and 6 μg pFLAG-cmv8 or pFLAG-cmv8-human ATG5/7 plasmids, respectively. To confirm CSP-induced ubiquitination of ATGs *via* upregulation of NEDD4, 2 × 106 293FT cells in two 6-wells were co-transfected with 2 μg His-UB, 4 μg pFLAG-cmv8 or pFLAG-cmv8-human ATG5/7, 2 μg shNC or sh*NEDD4*, and 2 μg pcDNA3.1 or pcDNA3.1-CSP-HA plasmids, respectively. To test the influence of the NLS domain on the CSP-induced ubiquitination of ATGs, 2 × 106 293FT cells in two 6-wells were co-transfected with 2μg His-UB plasmids, 4 μg pFLAG-cmv8-human ATG5/7, 4 μg pcDNA3.1, pcDNA3.1-CSP-HA, and pcDNA3.1-CSPΔNLS-HA, or pcDNA3.1-NLS-HA, respectively. Briefly, the cells were treated with 10 μM MG-132 for 24 h and lysed with 1 mL of western and IP cell lysis buffer. The protein concentrations of each group were determined and equalized as described above. Fusion FLAG-hATG5 and hATG7 proteins were captured using anti-FLAG ^®^ M2 Magnetic Beads (Sigma-Aldrich) according to the manufacturer’s instructions. After washing five times with equilibrium buffer (50 mM Tris HCl, 150 mM NaCl, pH 7.4), the beads were boiled with SDS-PAGE loading buffer for 10 min. An equal volume of total cell lysates (input) was loaded and separated by 10% SDS-PAGE, as described above. The PVDF filter was incubated with 1:500 mouse anti-FLAG tag (Invitrogen), 1:1000 rabbit monoclonal anti-His-Tag (Cell Signaling Technology), 1:2000 mouse anti-β-actin (Sigma-Aldrich), or 1:500 rabbit anti-NEDD4 overnight at 4°C and incubated with secondary antibody (Santa Cruz) for 1 h at room temperature. The protein bands were visualized using the Western BLoT Hyper HRP Substrate (TAKARA) and exposed using a chemiluminescence imaging system (Fusion Solo S, Vilber, France). Image J software (NIH) was used for grey value analysis.

### RNA Interference

RNA interference for ATG5 and ATG7 in HepG2 cells was performed according to the protocol of the ATG5/7 human shRNA plasmid kit (Origene, Rockville, MD, USA). HepG2 cells (1 × 10^6^) in 6-well plates were transfected with 4 μg ATG-interfering or scrambled non-effective plasmids using Lipofectamine 3000 following the manufacturer’s protocol. The plasmids containing the following sequences were used for ATG5 mRNA interference: 5-TCAGCTCTTCCTTGGAACATCACAGTACA-3, ATG7 mRNA interference, 5-CTTGGCTGCTACTTCTGCAATGATGTGGT-3, and shNC sequence, 5-GCACTACCAGAGCTAACTCAGATAGTACT-3’. For NEDD4 mRNA interference, 5-GTGAAATTGCACATAATGAGGTTCAAGAGACCTCATTATGTGCAATTTCAC-3, and shNC sequence, 5-GTTCTCCGAACGTGTCACGTCAAGAGATTACGTGACACGTTCGGAGAA-3′. Fresh culture medium containing 3 μg/mL puromycin (Gibco) was added to select puromycin-resistant cells. RNA interference efficiency was validated using quantitative real-time PCR and western blotting.

### Transcriptome Sequencing

CSP-stably transfected or control cells (1 × 10^6^) were lysed with 1 mL Trizol (Invitrogen), and total RNA was extracted as described above. A total of 2 μg RNA per sample was used as the input material for the RNA sample preparations. mRNA was purified from total RNA using poly T oligo-attached magnetic beads. Fragmentation was carried out using divalent cations under elevated temperatures in the VAHTSTM First Strand Synthesis Reaction Buffer (5X). First-strand cDNA was synthesized using a random hexamer primer and M-MuLV reverse transcriptase (RNase H). Second-strand cDNA synthesis was subsequently performed using DNA polymerase I and RNase H. Remaining overhangs were converted into blunt ends *via* exonuclease/polymerase activities. After adenylation of the 3’ ends of the DNA fragments, an adaptor was ligated for library preparation. To select cDNA fragments of preferentially 150–200 bp in length, the library fragments were purified with the AMPure XP system (Beckman Coulter, Beverly, USA). Then, 3 μL USER Enzyme (NEB, Ipswich, MA, UK) was incubated with size-selected, adaptor-ligated cDNA at 37°C for 15 min followed by 5 min at 95°C before PCR. PCR was performed with Phusion High-Fidelity DNA polymerase, Universal PCR primers, and Index (X) Primer. Finally, PCR products were purified (AMPure XP system) and library quality was assessed using the Agilent Bioanalyzer 2100 system. Libraries were quantified and pooled. Paired-end sequencing of the library was performed using HiSeq XTen sequencers (Illumina, San Diego, CA). RAW data were submitted to the GEO database, and the series records were GSE129323. A heatmap was generated using R language version 4.0.2, and the pheatmap package v1.0.12.

### Dual Luciferase Reporter Assay

Promoter activity assay was conducted by co-transfecting 100 ng pcDNA3.1 vector, pcDNA3.1-CSP-HA, pcDNA3.1-CSPΔNLS-HA or pcDNA3.1-NLS-HA, and 100ng pGL3-NEDD4 promoter and 1ng RL-TK into HEK293T cells in a 96-well 24 h later. The cells were then lysed and luciferase activity for each group was detected using the Dual-Luciferase^®^ Reporter Assay System (Promega, Madison, WI, USA).

### Statistical Analysis

SPSS software (version 19.0; IBM, Armonk, NY, USA) was used for statistical analysis. Student’s t-test for two groups or one-way ANOVA for multiple groups were used if the data were normally distributed to compare continuous variables, and if not, the Mann-Whitney U test was used for comparisons among groups. Pairwise differences in normally distributed variables were compared using the Tukey-Kramer statistic for multiple comparisons; if not, Dunnett’s test was used. Statistical significance was set at P < 0.05. Error bars represent standard deviations of the mean.

## Results

### CSP Translocated Into Cytoplasm of Hepatocyte Mediates the Resistance to the IFN-γ-Mediated Killing of EEFs

**Figure 1:**
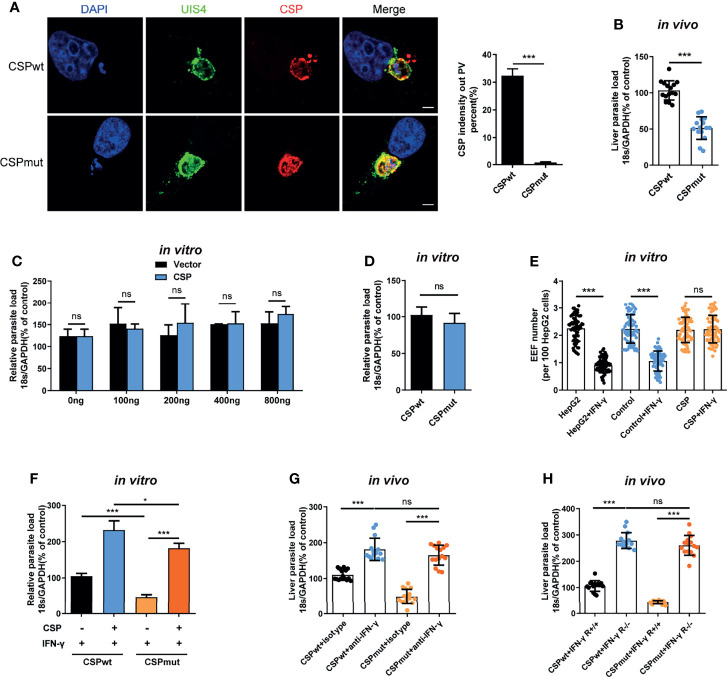
CSP translocated into cytoplasm of hepatocyte mediates the resistance to the killing of EEFs by IFN-γ. **(A)** After 1.2 × 10^5^ HepG2 cells were incubated with 4 × 10^4^ CSP_wt_ and CSP_mut_ *P.b* ANKA sporozoites for 24 h, then cells were stained with anti-UIS4, anti-CSP and DAPI. Translocation of CSP of EEFs (n=60) from the PV into HepG2 cytoplasm was observed under confocal microscopy (*left*), and quantified (*right*). Scale bar =5 μm. **(B)** After the mice were intravenously injected with 1,000 CSP_wt_ or CSP_mut_ parasite sporozoites for 46 h, the parasite burden in the liver was measured as the ratio of *Plasmodium* 18S rRNA to mouse GAPDH using Taqman real-time PCR. Each dot represents one mouse (n=15). **(C)** 1.2 × 10^5^ HepG2 cells were transiently transfected with the indicated amount of CSP plasmid and then incubated with 4 × 10^4^ CSP_wt_ sporozoites for 46 h; the parasite burden was determined as the ratio of *Plasmodium* 18S rRNA to human GAPDH using Taqman real-time PCR. Three individual experiments have been performed. **(D)** After 1.2 × 10^5^ HepG2 cells were incubated with 4 × 10^4^ CSP_wt_ or CSP_mut_ sporozoites for 46 h, the parasite burden was determined as described in **(C)**. The experiments have been performed for three times. **(E)** 1.2 × 10^5^ HepG2 (without lentivirus infection), control (infected with Lenti-pCDH control lentivirus and selected by puromycin) and CSP-stably transfected HepG2 cells were pre-treated with or without 1 U/mL IFN-γ, and then incubated with 4 × 10^4^ CSP_wt_ sporozoites for 46 h. EEF number per 100 HepG2 cells was determined and statistically analyzed. Each dot represents one visual field (n=66, magnification, 630×). **(F)** 1.2 × 10^5^ control and CSP-stably transfected HepG2 cells were treated with 1 U/mL IFN-γ, and then infected with CSP_wt_ or CSP_mut_ parasite as above; 46 h later, the parasite burden in hepatocytes was determined and compared as described in **(C)**. Three individual experiments have been performed. **(G)** Mice pre-treated with anti-IFN-γ or isotype antibody, were infected with 1,000 CSP_wt_ or CSP_mut_ sporozoites. Then, the liver burden of parasite at 46h post infection was determined as described in **(B)**. Each dot represents one mouse (n=15). **(H)** IFN-γR1 knockout and WT mice were infected with 1,000 CSP_wt_ or CSP_mut_ sporozoites, and liver burden was determined at 46 h after infection as described as above. Each dot represents one mouse (n=15). The pooled data of three repeated experiments was presented, data are represented as mean ± SD and analyzed by a student’s t-test or One-Way ANOVA. ns, not significant; *P < 0.05; ***P < 0.001.

The pexel I-II domain of CSP has been demonstrated to mediate its translocation from the PV into the hepatocyte cytoplasm (11). To test whether CSP could facilitate EEF survival in the PV of hepatocytes, a CSP pexel I-II domain mutant *Plasmodium berghei* (*P.b*) ANKA, named after CSP_mut_, was constructed by replacing the wild-type (WT) *CSP* with a pexel I-II mutant *CSP* using CRISPR-Cas9 technology (**Supplementary Figures S1A**–**C**). As no mutations were found in any of the potential off-target regions screened by genome-wide comparison (**Supplementary Table S1**), the potential of genomic off-target mutations generated by the CRISPR-Cas9 strategy could be excluded. The growth of the blood-stage mutant parasite was normal as compared to that of the WT parasite (CSP_wt_), and the salivary gland sporozoites of CSP_mut_ could be successfully generated after the mutant parasite-infected mosquitoes (**Supplementary Figure S1D**). Meanwhile, no difference of invasion ability into hepatocytes was found between CSP_mut_ and CSP_wt_ parasites (4.3 ± 0.2% vs 4.0 ± 0.1%) at 6 h post infection *in vitro*. Immunofluorescence assay with anti-UIS4, a parasitophorous vacuole membrane (PVM) specific marker and anti-CSP demonstrated that the CSP of mutant sporozoites was significantly inhibited to translocate from the PV into the hepatocyte cytoplasm when infecting HepG2 cells (**Figure 1A**).

Consistent with a previous study (11), we also found that the burden of CSP_mut_ in the liver was greatly reduced compared to that of the CSP_wt_ after the mice were infected (**Figure 1B**). However, transient transfection with full-length CSP plasmid did not enhance the development of EEFs *in vitro*, even when up to 800 ng of plasmids were transfected (**Figure 1C**). There was also no difference in parasite load in HepG2 cells infected with CSP_wt_ or CSP_mut_ parasites *in vitro* (**Figure 1D**). As sporozoite infection could trigger mice to generate immune responses against parasites (4), the different results between our *in vivo* and *in vitro* assays might be interpreted by the *in vivo* immune response that was not considered in our *in vitro* assay. Thus, we postulated that CSP might indirectly promote EEF development by suppressing host immune responses.

*Plasmodium* in the liver can trigger a type I interferon (IFN) response and recruit CD1d-restricted NKT cells (5). IFN-γ, predominately secreted by recruited NKT cells, is considered to be not only the major innate immune effector to suppress the development of the liver stage *in vivo* (5, 13), but also the critical effector to inhibit the EEFs in the PV of hepatocytes *in vitro* (14, 15). Altogether, these findings strongly suggest that IFN-γ should be included in our investigation of the effects of CSP on EEF development both *in vitro* and *in vivo*. Strikingly, we found that overexpression of CSP greatly inhibited the IFN-γ-mediated killing of EEFs, as both EEF number and parasite load in CSP-stably transfected HepG2 cells were much higher than those in control cells, when treated with IFN-γ (**Figure 1E** and **Supplementary Figure S2**). In addition, although the mutant parasite in HepG2 cells was susceptible to IFN-γ-mediated killing, overexpression of CSP remarkably reversed the killing effect of IFN-γ (**Figure 1F**). Subsequently, this finding was further confirmed in a physiologically relevant context, as no substantial difference in the liver parasite burden was found between the CSP_wt_ and CSP_mut_-infected mice when IFN-γ was depleted (**Figure 1G**). Similar results were obtained when two parasite strains were infected with IFN-γR1 knockout mice (**Figure 1H**). The reduced parasite burden in CSP_mut_ infected mouse livers was not due to the capacity of mutant parasites to induce the host to produce a much higher level of IFN-γ because a comparable mRNA level of IFN-γ was found in the livers of mice infected with CSP_wt_ or CSP_mut_ sporozoites at different doses (**Supplementary Figure S3**). Thus, our data demonstrates that translocated CSP from the PV into the hepatocyte cytoplasm confers resistance to IFN-γ-mediated killing of EEFs.

### Nitric Oxide (NO) Is Not Involved in the Suppression of the IFN-γ-Killing of EEFs Mediated by CSP

Previous studies have shown that IFN-γ prevents *Plasmodium* liver-stage development by inducing the expression of inducible nitric oxide synthase (iNOS), an enzyme required for the production of NO (6, 16, 17). Hence, we sought to investigate whether inhibition of the IFN-γ-mediated killing of EEFs by CSP was dependent on the downregulation of iNOS and NO. We found that transfection with CSP did not reduce the mRNA level of iNOS or the concentration of NO in the infected-HepG2 cells treated with IFN-γ (**Supplementary Figure S4A**). Similarly, both the levels of iNOS and NO were comparable between CSP_wt_ and CSP_mut_ parasite-infected HepG2 cells treated with IFN-γ (**Supplementary Figure S4B**). In addition, the resistance to the IFN-γ-mediated suppression of EEFs and the production of NO in CSP-transfected HepG2 cells treated with IFN-γ were not affected by treatment with two iNOS inhibitors, aminoguanidine (AG) and L-NAME. Although they could greatly inhibit the production of NO in lipopolysaccharide (LPS)-stimulated macrophages (**Supplementary Figure S4C**). Therefore, NO is not involved in the suppression of IFN-γ-mediated killing of EEFs by CSP.

### Autophagy and Its Related Proteins Are Essential for the IFN-γ-Mediated Killing of EEFs

**Figure 2:**
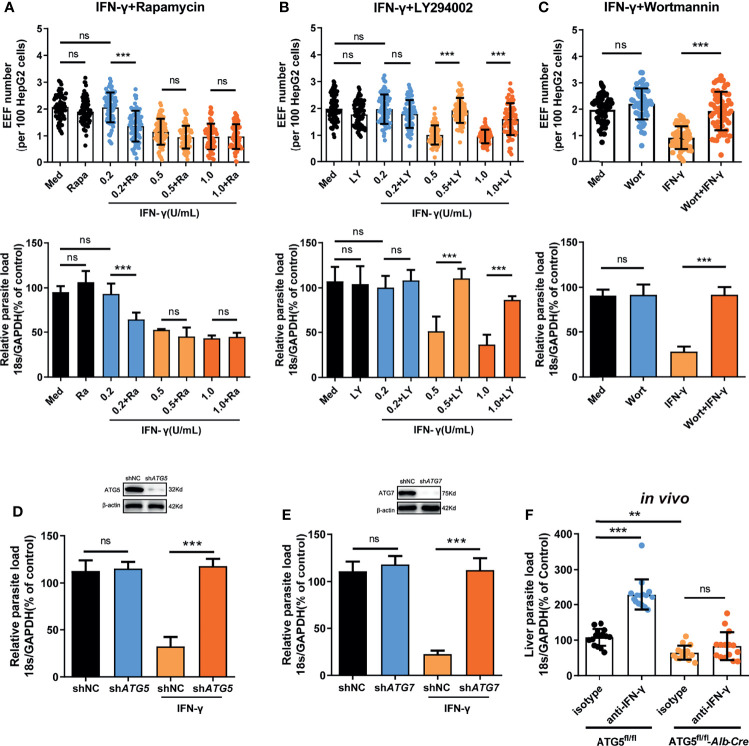
Autophagy and ATGs are essential for the IFN-γ-mediated killing of EEFs. **(A)** 1.2 × 10^5^ HepG2 cells were pre-treated with or without the autophagy inducer rapamycin (Ra) and indicated concentrations of IFN-γ and then incubated with 4 × 10^4^ CSP_wt_ sporozoites. The EEF number (*up*, each dot represents one visual field, n=75, magnification, 630×) and the parasite load (*bottom*) at 46 h post-infection were determined and compared. Three individual experiments have been performed. Med, medium. **(B)** HepG2 cells were pre-treated with or without the autophagy inhibitor LY294002 (LY) and IFN-γ at the indicated concentrations and then incubated with CSP_wt_ sporozoites as above. The EEF number (*up*, each dot represents one visual field, n=75, magnification, 630×) and the parasite load (*bottom*) at 46 h post-infection were determined and compared. The experiment has been repeated for three times. Med, medium. **(C)** HepG2 cells were pre-treated with or without the autophagy inhibitor wortmannin (Wort) at 100 mM and 1 U/mL IFN-γ, and then incubated with CSP_wt_ sporozoites as above for 46 h. The EEF number (*up*, each dot represents one visual field, n=60, magnification, 630×) and the parasite burden (*bottom*) at 46 h post-infection were determined and compared. Three individual experiments have been performed. Med, medium. **(D)** HepG2 cells were transiently transfected with shNC or sh*ATG5* plasmids, and then treated with 1 U/mL IFN-γ and infected with CSP_wt_ sporozoites as above for 46 h. The knockdown of ATG5 was verified by western blot (*up*), and the parasite burden (*down*) in HepG2 cells was determined and compared. Three individual experiments have been performed. **(E)** HepG2 cells were transiently transfected with shNC or sh*ATG7* plasmids and then treated with 1 U/mL IFN-γ and infected with CSP_wt_ sporozoites as above for 46 h. The knockdown of ATG7 was verified by western blotting (*up*), and the parasite burden (*down*) in HepG2 cells was determined and compared. The experiment has been performed for three time. **(F)** After IFN-γ was depleted with or without anti-IFN-γ, both control (ATG5^fl/fl^) and ATG5^fl/fl^-*Alb*-*Cre* mice were intravenously injected with 1,000 CSP_wt_ sporozoites, 46h later, the liver parasite burdens were measured and compared. Each dot represents one mouse (n=15). The pooled data of three repeated experiments was presented, data are represented as mean ± SD and analyzed by One-Way ANOVA and analyzed by One-Way ANOVA. ns, not significant; **P < 0.01; ***P < 0.001.

Evidence has shown that autophagy and autophagy-related genes (ATGs) are essential for the IFN-γ-mediated killing of *Toxoplasma gondii* in macrophages (18–21), an apicomplexan parasite closely related to *Plasmodium*. Thus, we next sought to investigate whether autophagy was also involved in the regulation of IFN-γ-mediated killing of EEFs in HepG2 cells. Consistent with our previous study (22), autophagy alone, either enhanced by rapamycin or inhibited by LY294002, had no effect on liver stage development in HepG2 cells (**Figures 2A, B**). However, rapamycin-enhanced autophagy significantly reduced the EEF number, parasite load, and EEF size in HepG2 cells in the presence of 0.2 U/mL IFN-γ (**Figure 2A** and **Supplementary Figure S5A**). We found that rapamycin could not further reduce the parasite number and load, as well as the size, when treated with 0.5 U/mL or 1 U/mL IFN-γ (**Figure 2A** and **Supplementary Figure S5A**), possibly because most of the EEFs were already killed by IFN-γ at these higher concentrations (14), and enhanced autophagy could not further increase the effect of IFN-γ. Meanwhile, the autophagy inhibitor LY294002 considerably reverted the killing effect of 0.5 U/mL and 1 U/mL IFN-γ on EEFs (**Figure 2B** and **Supplementary Figure S5B**), and a similar result was also obtained using another autophagy inhibitor, wortmannin (**Figure 2C**). No toxicity effect on cells was found for any of the above three drugs and IFN-γ at the working concentrations (**Supplementary Figure S5C**), excluding the possible effect of these drugs on EEF development in HepG2 cells. Furthermore, knockdown of either ATG5 (key component of the ATG12-ATG5 conjugate) or ATG7(E1-like enzyme), by specific small hairpin RNA (shRNA) could significantly inhibit the IFN-γ-mediated suppression of EEF development (**Figures 2D, E**).

In addition, the depletion of IFN-γ greatly increased the liver parasite load in control ATG5^fl/fl^ mice but had no effect on parasite burden in ATG5^fl/fl^-*Alb*-*Cre* mice, in which ATG5 in hepatocytes was specifically knocked out (**Figure 2F**), indicating the essential role of ATG5 in mediating the IFN-γ-killing of EEFs. However, we also noted that the level of liver burden in ATG5^fl/fl^-Alb-Cre mice was even lower than that in control ATG5^fl/fl^ mice in the presence of IFN-γ (**Figure 2F**). The reduced liver burden in ATG5^fl/fl^-*Alb*-*Cre* mice may be caused by completely abolishing of canonical autophagy after ATG5 knockout. As canonical autophagy could promote parasite development by supplying essential nutrients (23), and the lack of canonical autophagy greatly limited the replication of EEF in hepatocyte, even in the absence of IFN-γ. Thus, our findings show that both autophagy and ATGs are pivotal for the killing and suppression of EEF development in hepatocytes by IFN-γ, which is consistent with the report that an ATGs-mediated LAP-like process was also involved in the IFN-γ-mediated killing of the malaria liver stage (8).

### CSP Inhibits the IFN-γ-Mediated Suppression of EEFs by the Downregulation of ATGs

**Figure 3:**
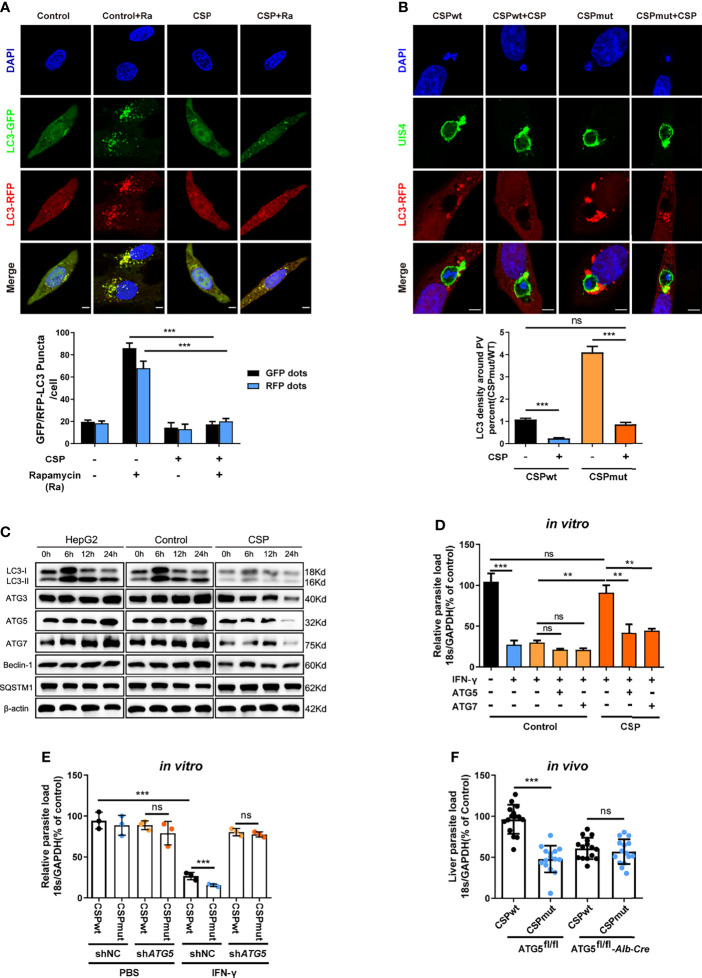
CSP inhibits the IFN-γ-mediated suppression of EEFs by the downregulation of ATGs. **(A)** 1.2 × 10^5^ control and CSP-stably transfected HepG2 cells were infected with Ad-mRFP-GFP-LC3 virus, and then treated with the autophagy inducer rapamycin for 24 h. Both LC3-RFP or LC3-GFP puncta in HepG2 cells were imaged (*up*) and qualified (*down*). Scale bar = 5 μm, n=60. **(B)** 1.2 × 10^5^ control and CSP-stably transfected HepG2 cells were transiently transfected with LC3-RFP plasmid, and then incubated with 4 × 10^4^ CSP_wt_ or CSP_mut_ sporozoites for 24 h. Cells were fixed and stained with anti-UIS4, and LC3 surrounding the EEFs was observed under confocal microscopy (*up*), and the intensity of LC3 around PV was quantified (*down*), Scale bar = 5 μm, n (EEFs) =60. **(C)** HepG2 (without lentivirus infection), control (infected with Lenti-pCDH control lentivirus and selected by puromycin) and CSP-stably transfected HepG2 cells were treated with rapamycin for the indicated times. Protein levels of ATGs, including LC3I/LC3II, ATG3, ATG5, ATG7, Beclin-1, and p62 (SQSTM1), was determined by western blotting. **(D)** Control and CSP-stably transfected HepG2 cells transfected with or without ATG5, or ATG7 plasmid, and then treated with 1 U/mL IFN-γ and infected with CSP_wt_ sporozoites as above for 46 h. The parasite load in HepG2 cells was determined and compared. Three individual experiments have been performed. **(E)** HepG2 cells were transiently transfected with Scramble or ATG5 shRNA plasmids, and then treated with 1 U/mL IFN-γ and incubated with CSP_wt_ or CSP_mut_ sporozoites as above. 46 h later, the parasite load in the HepaG2 cells was determined and compared. The experiment has been performed for three times. **(F)** Both control (ATG5^fl/fl^) and ATG5^fl/fl^-*Alb*-*Cre* mice were infected by intravenous injection with 1,000 CSP_wt_ or CSP_mut_ sporozoites, 46 h later, the liver burden of the two parasites in control and ATG5^fl/fl^-*Alb*-*Cre* mice were determined and compared. Each dot represents one mouse, n=15. The pooled data of three repeated experiments was presented, data are represented as mean ± SD, and analyzed by a student’s t-test, Mann-Whitney U test or One-Way ANOVA; ns, not significant; **P < 0.01; ***P < 0.001.

We then investigated whether CSP could inhibit autophagy and the expression of ATGs. Immunofluorescence assay showed that rapamycin-induced autophagy was significantly inhibited in CSP-stably transfected HepG2 cells as either the fluorescence intensity of the key autophagy marker, microtubule-associated protein 1 light chain 3 (LC3)-RFP or LC3-GFP puncta, induced by rapamycin, was reduced by more than 3 folds in CSP-stably transfected HepG2 cells as compared to that in control cells (**Figure 3A**). In addition, the intensity of LC3 fluorescence around the CSP_mut_ EEFs was much stronger than that of CSP_wt_ EEFs in HepG2 cells, and the level of LC3 fluorescence intensity around CSP_mut_ EEFs was comparable to that around CSP_wt_ EEFs in CSP-overexpressing HepG2 cells (**Figure 3B**). Next, the effect of CSP on the expression of ATGs was investigated. LC3 levels were significantly reduced in CSP-stably transfected HepG2 cells treated with rapamycin at all test time points when compared with control group, and the levels of autophagy-related genes, including Beclin-1, ATG3, ATG5, and ATG7, were also significantly decreased at 24 h. In contrast, the autophagy adaptor protein SQSTM1 (p62) significantly accumulated in CSP-stably transfected HepG2 cells, indicating the suppression of autophagy (**Figure 3C** and **Supplementary Figure S6**). Overall, these findings demonstrate that CSP could inhibit autophagy and the expression of ATGs.

As CSP could significantly suppress autophagy and its related proteins, we next investigated whether the resistance of CSP to IFN-γ-mediated suppression of EEFs was closely associated with its ability to suppress ATGs expression. Although the CSP-stably transfected HepG2 cells had a significant resistance to the IFN-γ-mediated suppression of EEFs, the overexpression of either ATG5 or ATG7 significantly reduced this inhibitory effect (**Figure 3D**). Similarly, the CSP_wt_ and CSP_mut_ parasite burdens in ATG5-knockdown HepG2 cells were comparable when the cells were treated with IFN-γ (**Figure 3E**). To further confirm this finding, both control (ATG5^fl/fl^) and ATG5^fl/fl^-*Alb*-*Cre* mice were infected with either CSP_wt_ or CSP_mut_ sporozoites, and as expected, there was no difference in the liver burdens in ATG5^fl/fl^-*Alb*-*Cre* mice between the two parasites (**Figure 3F**). Taken together, these findings demonstrate the essential role of ATGs in the resistance to IFN-γ-mediated suppression by CSP.

### The CSP-Mediated Resistance of IFN-γ- Killing of EEFs Is Dependent on Its Ability to Enhance ATGs Ubiquitination

**Figure 4:**
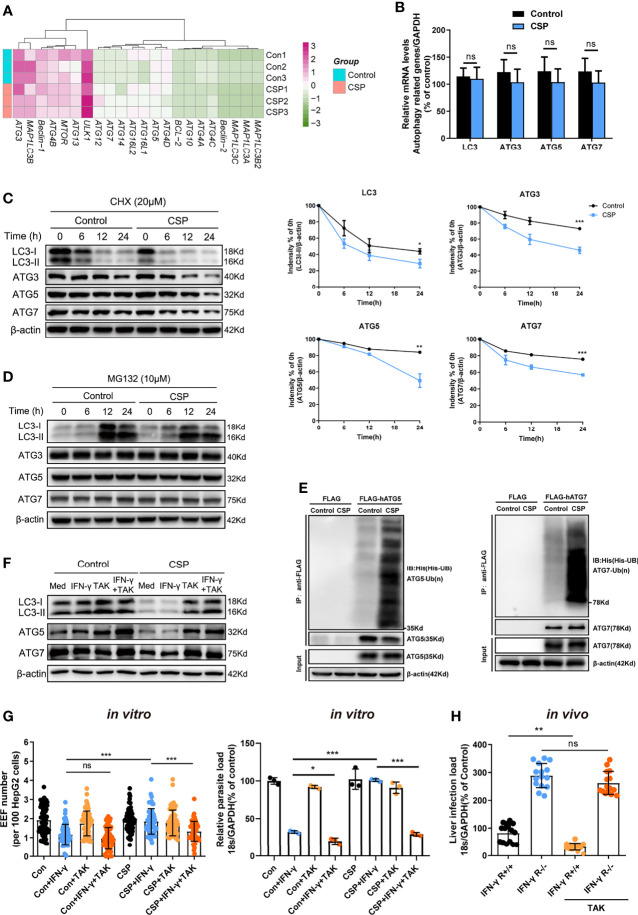
The CSP-mediated resistance to IFN-γ- killing of EEFs through the enhance of ATGs ubiquitination. **(A)** The mRNA levels of ATGs between control and CSP-stably transfected HepG2 cells in the heatmap were compared, and three biological repeats were performed. **(B)** The different mRNA levels of ATGs (ATG3, ATG5, ATG7, and LC3) between control and CSP-stably transfected HepG2 cells were compared using real-time PCR. Three individual experiments have been performed**. (C)** Control and CSP-stably transfected HepG2 cells were treated with CHX for the indicated times, and the protein levels of LC3I/LC3II, ATG3, ATG5 and ATG7 were detected by western blotting (*left*); the relative expression levels of LC3I/LC3II, ATG3, ATG5, and ATG7 to β-actin were quantified (*right*). Three individual experiments have been performed. **(D)** Control and CSP-stably transfected HepG2 cells were treated with the proteasome inhibitor MG132 for the indicated times, and the protein levels of LC3I/LC3II, ATG3, ATG5, and ATG7 were detected by western blot. The experiment have been performed for three times. **(E)** Ubiquitination assay showed that CSP promoted the ubiquitination of ATG5 (*left*) and ATG7 (*right*). Control and CSP-stably transfected HepG2 cells were transfected with His-UB, and FLAG-hATG5 or FLAG-hATG7 plasmids, and treated with MG-132 for 24 h. Then, cells were collected for immunoprecipitation of FLAG-hATG5 and FLAG-hATG7 followed by immunoblot analyses of ubiquitin (His-UB). Cells transfected with FLAG vector was used as negative controls. Total cell lysates (input) were also immunoblotted with antibodies against FLAG and β-actin. The experiment has been repeated for three times. **(F)** The levels of protein LC3, ATG5 and ATG7 were determined by western blotting in both control and CSP-stably transfected HepG2 treated with or without 1μM TAK (TAK-243) and 1 U/mL IFN-γ for 24 h. **(G)** 1.2 × 10^5^ CSP-stably transfected HepG2 or control cells were treated with or without 1μM TAK (TAK-243), and then were treated with 1 U/mL IFN-γ and infected with 4 × 10^4^ CSP_wt_ sporozoites for 46 h. The EEF number (*left*, each dot represents one visual field, n=57-73, magnification, 630×) and parasite load (*right*) were determined and compared as above. Three individual experiments have been performed **(H)** IFN-γ R1 knockout and WT mice were per-treated with or without 20mg/kg TAK-243 and infected with 1,000 CSP_wt_ sporozoites in the next day, then liver parasite burden was determined at 46 h after infection as described as above. Each dot represents one mouse (n=15). The pooled data of three repeated experiments was presented, data are represented as mean ± SD and analyzed by a student’s t-test or One-way ANOVA; ns, not significant; *P < 0.05; **P < 0.01; ***P < 0.001.

To explore the underlying mechanism by which CSP downregulates the expression of ATGs, mRNAs from CSP-stably transfected and control HepG2 cells treated with rapamycin were sequenced and compared. Unexpectedly, there was no significant downregulation in the mRNA levels of most ATGs in CSP-stably transfected HepG2 cells as compared to that of the control (**Figure 4A**), which was further confirmed by real-time PCR (**Figure 4B**). As CSP downregulated the protein levels of ATGs, this finding suggests that CSP does not regulate the expression of ATGs at the transcriptional level.

Eukaryotic cells use the autophagy-lysosome and ubiquitin-proteasome system as their major intracellular protein degradation pathways (24). However, autophagy level was significantly reduced in the CSP-stably transfected HepG2 cells (**Figure 3A**), indicating that the autophagy-lysosome system may not be responsible for the downregulation of the ATGs protein levels. Therefore, we next investigated whether CSP could downregulate the expression of ATGs at the post-translational level through the ubiquitin-proteasome pathway, which is a major intracellular degradation pathway and a widespread post-translational modification (25). The half-lives of LC3, ATG3, ATG5, and ATG7 in the CSP-stably transfected HepG2 cells were significantly reduced compared to those of the control, when cells were treated with the protein synthesis inhibitor cycloheximide (CHX) (**Figure 4C**). In addition, the levels of LC3, ATG3, ATG5, and ATG7 in CSP-stably transfected and control HepG2 cells were comparable when the cells were treated with the specific proteasome inhibitor MG132 (**Figure 4D** and **Supplementary Figure S7**). These findings indicate that CSP could regulate the degradation of ATGs in a proteasome-dependent manner. To investigate whether the degradation of ATGs in the proteasome was dependent on ubiquitination, the CSP-stably transfected and control cells were transfected with His-UB, FLAG-hATG5 (human ATG5), or FLAG-hATG7 (human ATG7) plasmids, and co-immunoprecipitation was performed using anti-FLAG beads to detect ubiquitin bound to ATGs. The ubiquitination assay showed that CSP promoted the ubiquitination of both ATG5 and ATG7 in HepG2 cells (**Figure 4E**).

To verify that the resistance of CSP to IFN-γ-mediated suppression of EEF development was due to the enhancement of ATGs ubiquitination, a ubiquitination-specific inhibitor TAK-243 (26), was used at non-toxic concentrations *in vitro* and *in vivo* (**Supplementary Figure S8**) to suppress ubiquitination. We found that treatment with TAK-243 significantly elevated the protein levels of ATGs (**Figure 4F**) and enhanced the IFN-γ-mediated killing of EEFs in CSP-stably transfected HepG2 cells, as compared to those in control cells (**Figure 4G**). Furthermore, the administration of TAK-243 greatly reduced the liver parasite burden in WT mice but had no significant effect on liver parasite burden in IFN-γ R1 knockout mice (**Figure 4H**). Thus, we demonstrate that CSP mediated resistance to IFN-γ- killing of EEFs through the upregulation of specific ubiquitination of ATGs.

### CSP Upregulates the E3 Ubiquitin Ligase NEDD4 to Promote the Ubiquitination of ATGs

**Figure 5:**
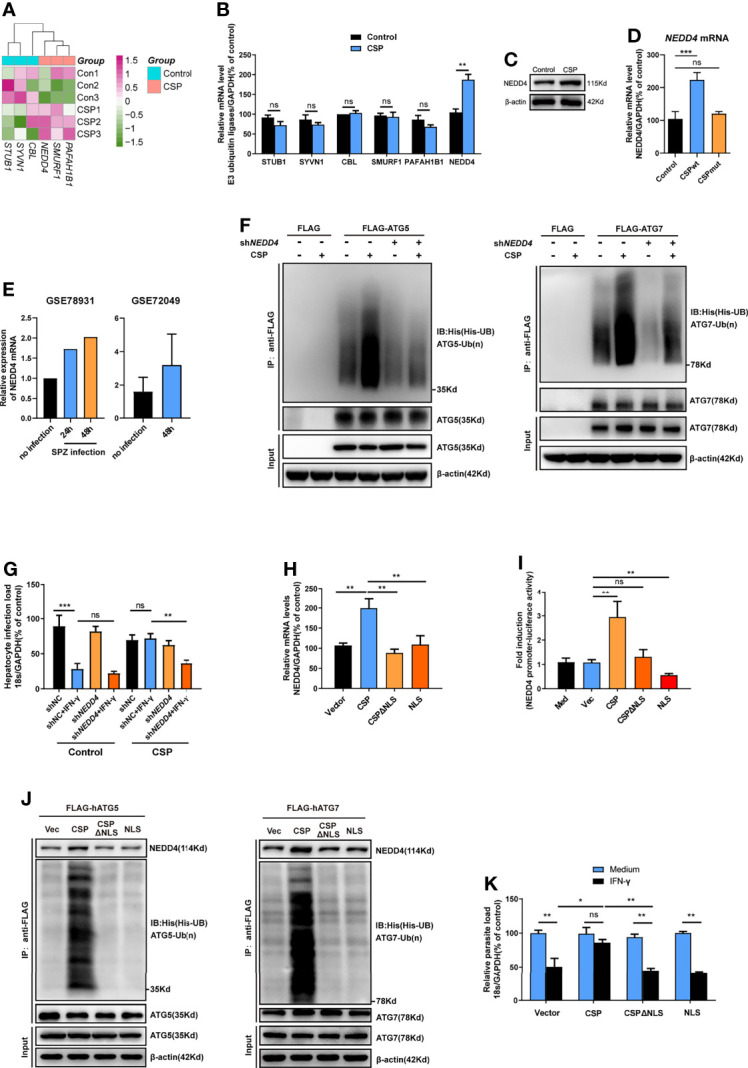
CSP upregulates the E3 ubiquitin ligase NEDD4 to promote the ubiquitination of ATGs. **(A)** The mRNA levels of six E3 ubiquitin ligases between control and CSP-stably transfected HepG2 cells in the heatmap were compared, and three biological replicates were performed. **(B)** The different mRNA levels of six E3 ubiquitin ligases in both control and CSP-stably transfected HepG2 cells at 24 after culture were confirmed by real-time PCR. Three individual experiments have been performed. **(C)** The protein levels of NEDD4 in both control and CSP-stably transfected HepG2 cells at 24 after culture were confirmed by western blotting. Three individual experiments have been performed. **(D)** After HepG2 cells were infected with CSP_wt_ or CSP_mut_ sporozoites as described as before for 24 h, the mRNA level of *NEDD4* was determined by real-time PCR, uninfected HepG2 cells were used as control. The experiment has been repeated for three times. **(E)** Relative level of *NEDD4* mRNA in *P.b* ANKA sporozoite infected Huh7 and HepG2 cells were analyzed based on data extracted from GSE78931 and GSE72049. **(F)** Ubiquitination assay showed that sh*NEDD4* deceased CSP-induced hATG5 (*left*) and hATG7 (*right*) ubiquitination in 293FT cells. 293FT cells were transfected with His-UB, pcDNA3.1 vector or pcDNA3.1-CSP, shNC or sh*NEDD4*, and FLAG vector, FLAG-hATG5 or FLAG-hATG7 plasmids, and treated with MG-132 for 24 h. Then, cells were collected for immunoprecipitation of FLAG-hATG5 and FLAG-hATG7 followed by immunoblot analyses of ubiquitin (His-UB). Cells transfected with FLAG vector was used as negative controls. Total cell lysates (input) were also immunoblotted with antibodies against FLAG and β-actin. Three individual experiments have been performed. **(G)** 1.2 × 10^5^ control and CSP-stably transfected HepG2 cells were transfected with shNC or sh*NEDD4* and treated with 1 U/mL IFN-γ or not, then infected with 4 × 10^4^ CSP_wt_ sporozoites, for 46 h. The parasite load in HepG2 cells was determined as above. The experiment has been performed for three times. **(H)** HepG2 cells were transfected with vector (Vec), CSP, CSPΔNLS, or NLS plasmids for 24 h, and the transcription of NEDD4 was determined by real-time PCR. Three individual experiments have been performed. **(I)** HEK293T cells were transfected with RL-TK, NEDD4 promoter reporter plasmid, and with or without Vec, CSP, CSPΔNLS, or NLS plasmids for 24 h, and the ratio of firefly luciferase to Renilla luciferase were determined using Dual Luciferase Assay kit. Three individual experiments have been performed. Med, medium. **(J)** The ubiquitination assay showed that deletion of NLS domain (ΔNLS) deceased CSP-induced hATG5 (*left*) and hATG7 (*right*) ubiquitination in 293FT cells. 293FT cells were transfected His-UB, and Vec, CSP, CSPΔNLS or NLS, and FLAG-hATG5 or FLAG-hATG7 plasmids, and treated with MG-132 for 24 h. Then, cells were collected for immunoprecipitation of FLAG-hATG5 and FLAG-hATG7 followed by immunoblot analyses of ubiquitin (His-UB). Cells transfected with pcDNA3.1 vector was used as negative controls. Total cell lysates (input) were also immunoblotted with antibodies against FLAG and β-actin. The experiment has been repeated for three times. **(K)** 1.2 × 10^5^ HepG2 cells were transfected with Vec, CSP, CSPΔNLS, or NLS plasmids and treated with or without 1 U/mL IFN-γ, followed by the infection with 4 × 10^4^ CSP_wt_ sporozoites for 46 h. The parasite load in HepG2 cells was determined as above. Three individual experiments have been performed. The pooled data of three repeated experiments was presented (except **E**), data are represented as mean ± SD and analyzed by Mann-Whitney U test or One-way ANOVA. UB, ubiquitin; ns, not significant; *P < 0.05; **P < 0.01, ***P < 0.001.

E3 ubiquitin ligase, which transfers the ubiquitin of the E2 enzyme to its attached substrate, is the key enzyme in the ubiquitination process (25). Therefore, we hypothesized that E3 ubiquitin ligases may be involved in the ubiquitination of ATGs (27). Six candidate E3 ligases, including STIP1 homology and U box-containing protein 1 (STUB1), Synovial apoptosis inhibitor 1 (SYVN1), Cbl proto-oncogene (CBL), SMAD specific E3 ubiquitin protein ligase 1 (SMURF1), Platelet activating factor acetylhydrolase 1b regulatory subunit 1 (PAFAH1B1), and neural precursor cell expressed, developmentally down-regulated 4 (NEDD4), were predicted to regulate the ubiquitination of at least two ATGs using an online prediction tool (**Supplementary Figure S9** and **Table S2**) (27). However, the RNA-seq data showed that among these ligases, only *NEDD4* was significantly upregulated in CSP-stably transfected HepG2 cells (**Figure 5A**), and the increase in both the mRNA and protein levels of NEDD4 was confirmed by real-time PCR and western blotting, respectively (**Figures 5B, C**). Furthermore, infection with the CSP_wt_ parasite, but not the CSP_mut_ parasite, significantly elevated the mRNA level of *NEDD4* in HepG2 cells (**Figure 5D**), strongly suggesting that the translocation of CSP from the PV into the cytoplasm could upregulate the expression of E3 ubiquitin ligase NEDD4. Consistently, the upregulation of *NEDD4* in sporozoite-infected hepatocyte cell lines was also found in the *P. b* ANKA infection model (data from GSE78931 and GSE72049) (28) (**Figure 5E**). Moreover, knockdown of *NEDD4* by shRNA significantly reduced CSP-mediated ubiquitination of ATG5 and ATG7 (**Figure 5F**). In addition, the resistance of CSP-overexpression to IFN-γ-mediated killing of CSP_wt_ parasites was significantly reduced after *NEDD4* was silenced (**Figure 5G**). This finding strongly suggests that NEDD4 was essential for CSP-mediated resistance to IFN-γ suppression of EEFs.

A previous study showed that CSP contained a nuclear location signal (NLS) domain, which could import the CSP into the nucleus (11), and we also confirmed that the transfection of CSP, but not CSPΔNLS plasmid (CSP without NLS domain), could import into the nucleus of HepG2 cells (**Supplementary Figure S10**). To better elucidate the mechanism of the upregulation of NEDD4 by CSP, the effects of CSP, CSPΔNLS, and NLS (only NLS domain) expression on the transcription of *NEDD4* in HepG2 cells were determined. Although the transfection of CSP upregulated *NEDD4* expression, neither the transfection of CSPΔNLS nor NLS influenced the transcription of NEDD4 in HepG2 cells (**Figure 5H**). The dual luciferase reporter assay confirmed that only CSP significantly activated the NEDD4 promoter (**Figure 5I**). These data indicate that translocation into the nucleus was essential for CSP to modulate the transcription of *NEDD4*, but the modulatory motif might not exist in the sequence of CSPΔNLS. In addition, co-immunoprecipitation results showed that more NEDD4 proteins could directly bind to both ATG5 and ATG7 and increased the ubiquitination of these two proteins in CSP, but not in the vector, CSPΔNLS or NLS-transfected 293FT cells (**Figure 5J**), and only the transfection of CSP remarkably inhibited the IFN-γ-mediated suppression of EEFs in HepG2 cells (**Figure 5K**). Overall, these data suggest that NEDD4 was an E3 ubiquitin ligase that specifically mediated the ubiquitination of ATG5 and ATG7, and that CSP enhanced the ubiquitination of ATGs through the upregulation of NEDD4.

## Discussion

Considerable progress has been made in identifying the molecules of sporozoites involved in their selective attachment to hepatocytes and the formation of the PV after their invasion (1, 29–31); however, the underlying mechanism of the survival of parasites in the PV has thus far remained unknown. Here, we found that nuclear translocation of cytoplasmic CSP could mediate the resistance to IFN-γ-killing of EEFs and facilitate the survival of parasites in the PV.

CSP is a multifunctional protein of the malaria parasite that is not only critical for the invasion of sporozoites into hepatocytes but is also essential for sporozoite development in mosquitoes (32–38). Therefore, CSP is regarded as a leading candidate protective antigen (39), which is included in several subunit vaccines to induce CSP-specific antibody and/or CD8^+^T cell responses against sporozoites (40–46). Our finding of this new aspect of CSP to subvert the host innate immune response not only supports the important role of CSP as the dominant protective antigen but also sheds new light on a novel prophylactic strategy against the liver stage.

Recent studies have shown that parasites can shed host autophagic proteins from the PVM to escape the deleterious effects of the PAAR response (47) and UIS3 sequestered LC3 to suppress autophagy in EEFs (48). These findings support the existence of subversive strategies for EEFs against hepatocyte autonomous immunity. In this study, we found that CSP translocated from the PV into the cytoplasm of hepatocytes inhibited the IFN-γ-mediated killing of EEFs by downregulating the expression of ATGs by ubiquitination, revealing a novel strategy for the liver stage of malaria to subvert host innate immunity. However, a previous study suggested that liver-stage development enhanced by CSP was associated with its ability to suppress NF-κB activation in infected hepatocytes. Although the real reason is definitively unknown for the different mechanisms of CSP to promote EEF development between our study and previous studies, the possible interpretation might be the missed consideration of IFN-γ in the natural malaria infection in a previous study.

It is well known that IFN-γ-mediated killing of intracellular *T. gondii* is regulated by IFN-γ-inducible GTPase Irg6 and guanylate-binding protein (GBP)1 (49–51), whereas Irg6 is not involved in the IFN-γ-mediated killing of EEFs (49). Indeed, we found that the knockout of GBP-1 also had no effect on EEF development in HepG2 cells treated with IFN-γ (**Supplementary Figure S11**). Thus, the roles of GTPase and GBPs in the IFN-γ-mediated killing of EEFs might differ from those in *T*. *gondii* and should be investigated in further detail.

Recent studies have demonstrated that infection with sporozoites could induce both canonical autophagy and the PAAR response of hepatocytes to EEFs (9, 22). Although canonical autophagy promotes parasite development by supplying essential nutrients (23), the role of PAAR response in liver stage development is conflicting (9, 52). One possible reason is that conditional ATG knockout of non-permissive mouse embryonic fibroblasts (MEFs) has been used in previous studies (9, 52). In the present study, we found that knockdown of either ATG5 or ATG7 in HepG2 cells, the permissive cells of sporozoites, had no significant effect on EEF development (**Figures 2D, E**). In contrast, the induction of autophagy by rapamycin enhanced the killing effect of IFN-γ (**Figure 2A**), and autophagy inhibitors or knockdown of ATGs attenuated the killing of EEFs by IFN-γ (**Figures 2B**–**E**). This demonstrates the essential role of autophagy in the regulation of the IFN-γ-mediated killing of EEFs. Therefore, we showed that autophagy alone has no significant effect on EEF development *in vitro*, but it plays a pivotal role in the regulation of IFN-γ-mediated killing of EEFs.

**Figure 6:**
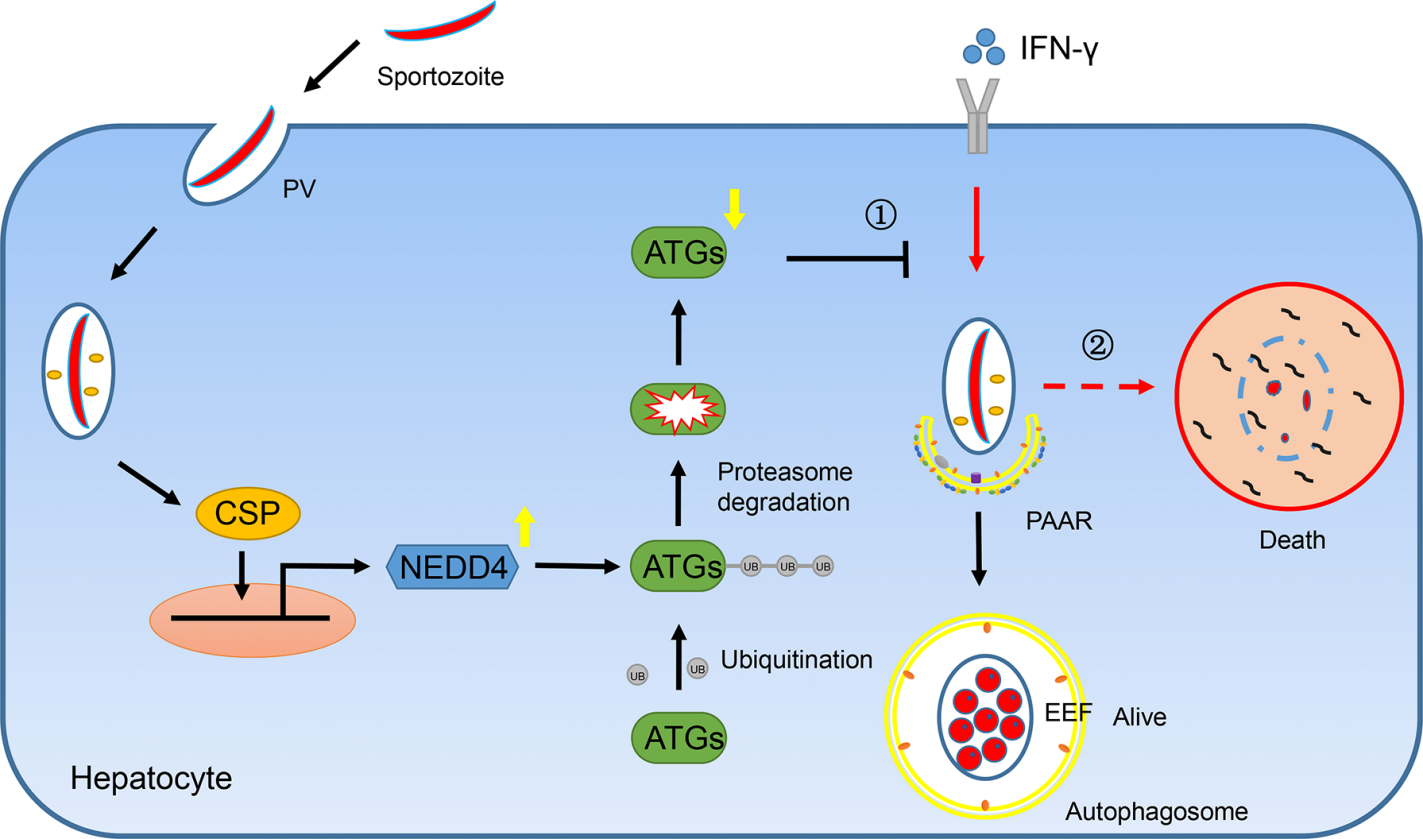
A mechanistic pathway of CSP-mediated resistance to IFN-γ- killing of EEFs in hepatocytes. ①After sporozoite invaded into hepatocytes, CSP on its surface would be translocated from PV into the cytoplasm of hepatocytes and is then introduced into nucleus dependent on its NLS domain. As a result, CSP activates the promoter of ligase3 NEDD4 and enhances its transcription. The upregulated NEDD4 promotes the ubiquitination of ATGs, leading to the degradation of ATGs. As ATGs are essential for IFN-γ-mediated killing of EEFs, the CSP-mediated resistance to IFN-γ, which is dependent on its ability to downregulate ATGs through enhancing NEDD4-mediated ubiquitination, facilitates the survival of EEFs. ②Otherwise, EEFs are destroyed by IFN-γ.

Taken together, we found that CSP translocated from the PV into the cytoplasm of hepatocytes could be imported into the nucleus and upregulated the transcription of E3 ligase NEDD4, depending on its NLS domain. The E3 ligase NEDD4 enhances the ubiquitination of ATGs, leading to a decrease in the protein level of ATGs in hepatocytes. As ATGs are essential for IFN-γ-mediated killing of EEFs, CSP resists the IFN-γ-mediated killing of EEFs and facilitates the survival of EEFs in hepatocytes (**Figure 6**). Thus, our findings explain why CSP translocated from the PV into the hepatocyte cytoplasm could promote liver stage development, and provide novel prophylaxis strategies to eliminate liver stage infection by chemically targeting CSP pexel, NLS domain, or ubiquitination in hepatocytes.

## Data Availability Statement

The datasets presented in this study can be found in online repositories. The names of the repository/repositories and accession number(s) can be found below: https://www.ncbi.nlm.nih.gov/geo/, GSE129323.

## Ethics Statement

The animal study was reviewed and approved by Animal Ethics Committee of Army Medical University.

## Author Contributions

Conceptualization, WX, YW, HZ, and JD. Writing – Original Draft, WX and HZ. Writing–Review & Editing, WX, YW, JD, and HZ. Methodology, HZ and XL. Investigation, HZ, XL, KL, KZ, CZ, TL, FZ, TZ, YD, and YF. Funding Acquisition, WX. Resources, WX, YW, and JD. Supervision, WX, YW, and JD. All authors contributed to the article and approved the submitted version.

## Funding

This work was supported by the National Natural Science Foundation of China (No. 81672053 and 81702247), the State Key Program of the National Natural Science Foundation of China (No. 81830067), and the Miaopu Talent Grant from Army Medical University (2019R057).

## Conflict of Interest

The authors declare that the research was conducted in the absence of any commercial or financial relationships that could be construed as a potential conflict of interest.

## Publisher’s Note

All claims expressed in this article are solely those of the authors and do not necessarily represent those of their affiliated organizations, or those of the publisher, the editors and the reviewers. Any product that may be evaluated in this article, or claim that may be made by its manufacturer, is not guaranteed or endorsed by the publisher.
