# Supplementary material for: ATG Ubiquitination Is Required for Circumsporozoite Protein to Subvert Host Innate Immunity Against Rodent Malaria Liver Stage
Source: Front Immunol. 2022 Feb 9;13:815936. doi: 10.3389/fimmu.2022.815936 (PMC8864237; doi:10.3389/fimmu.2022.815936)
Supplement: Supplementary file 1 [file DataSheet_1.docx]

Supplementary Material

# Supplementary Figures and Tables

## Supplementary Figures

**
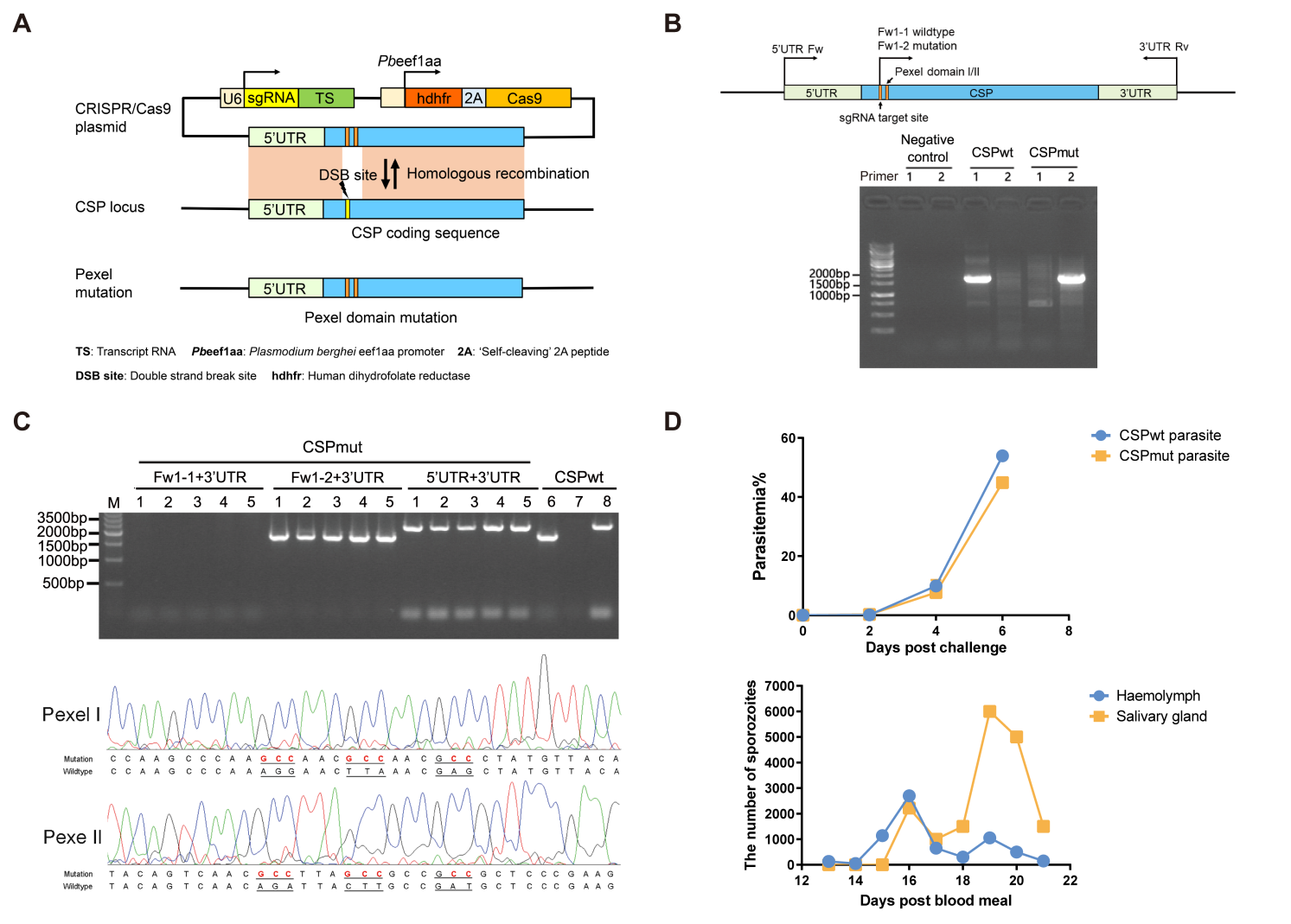
**

**Figure S1 Construction and characterization of the CSP_mut_ parasite.**

(**A**) Schematic construction of the CSP_mut_ parasite by CRISPR-Cas9. The plasmid contains Cas9 and sgRNA expression cassettes and donor template pexel/II mutant CSP for homologous recombination repair after a double-strand break (DSB) at the WT CSP. (**B**) Primers designed to identify the CSP_wt_ and CSP_mut_ parasite. A ~1.6-kb fragment was amplified from the genomic DNA of the mutant parasite with Primer 2 (Fw1-2 (Mut) and 3′-UTR Rv), but no fragment was obtained with Primer 1 (Fw1-1 (WT) and 3′-UTR Rv). (**C**) Identification of CSP_mut_ parasite clones. The pyrimethamine-resistant parasites in mice were collected and cloned by injecting each mouse with ~1.0 infected iRBCs. The resulting clones were identified by PCR (*top*) and the *CSP* gene was sequenced (*bottom*). (**D**) The parasitemia of mice infected with the CSP_mut_ or CSP_wt_ parasite was determined (*top*), sporozoites were examined in both the hemolymph and salivary gland of mosquitoes at indicated times post-infection with CSP_mut_ parasite (*bottom*).


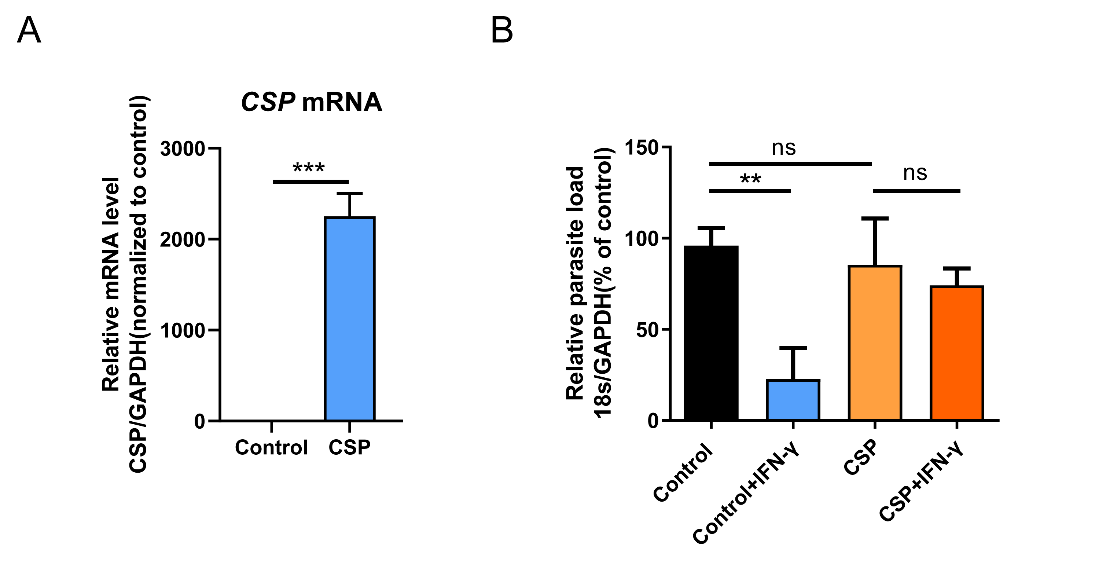


**Figure S2 The over-expression of CSP resists to the IFN-γ-mediated killing of EEFs.**

(A) The mRNA levels of CSP were evaluated by ER-qPCR in the Control and CSP-stably transfected HepG2 cells. GAPDH used as reference. (B) 1.2 × 10^5^ Control and CSP-transiently transfected HepG2 cells were treated with or without 1U/mL IFN-γ followed by incubation with 4 × 10^4^ CSP_wt_ sporozoites; 46 h later, the parasite burden was determined as described previously, n=3. Data are represented as mean ± SD and analyzed using the Mann-Whitney U test, the pooled data of three repeated experiments was presented. ns, not significant; ***p<*0.01; ****p<*0.001.


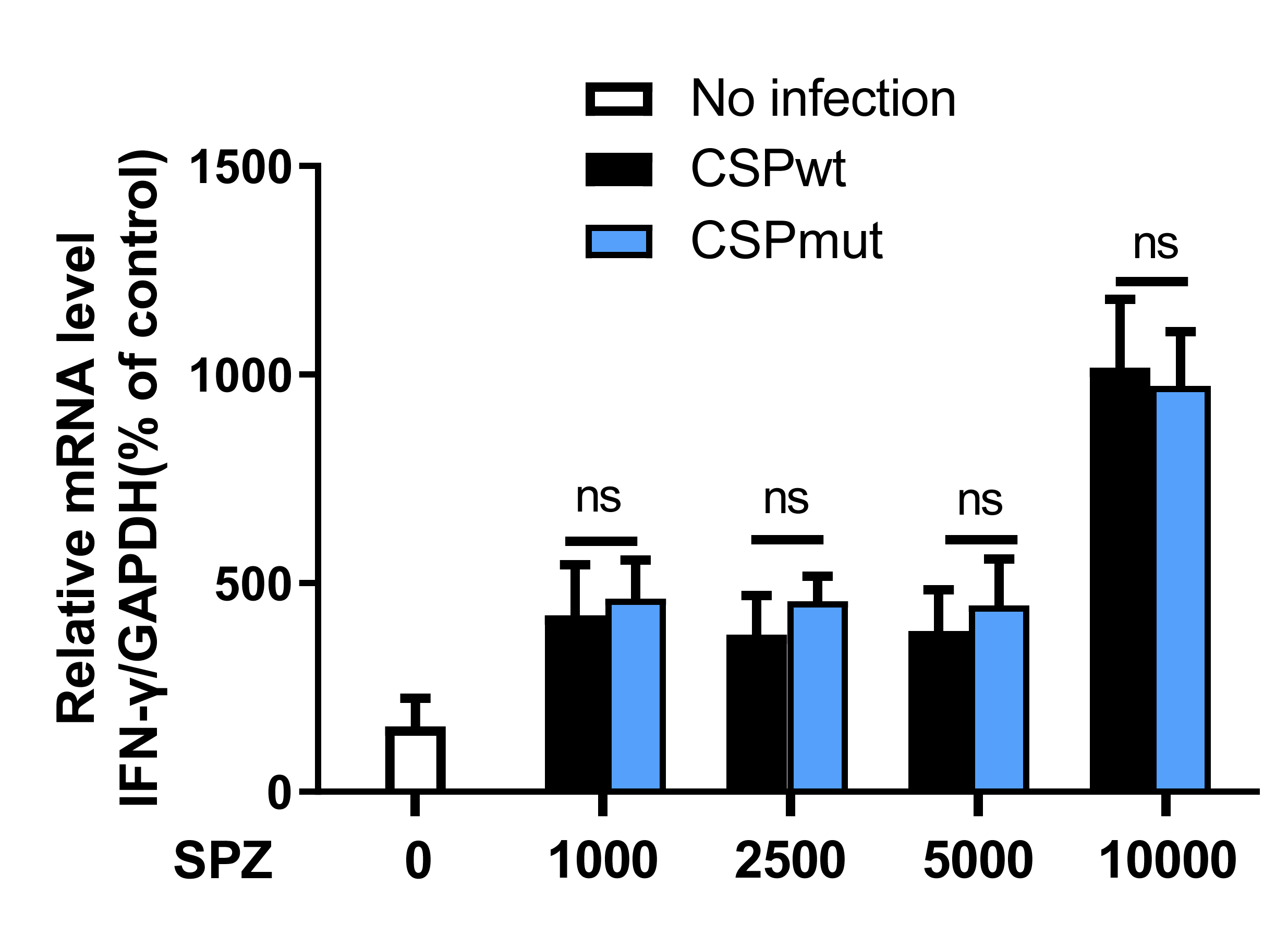


**Figure S3 The mRNA levels of IFN-γ in the liver of mouse after infected with CSP_wt_ or CSP_mut_ sporozoites.**

The mRNA levels of IFN-γ were evaluated in the liver mice (n=3) at 46 h after infection of indicated amount of CSP_wt_ or CSP_mut_ sporozoites by real-time PCR. Non-infected mice were the control. This experiment has been performed twice. Data are represented as mean ± SD and analyzed by Mann-Whitney U test; ns, not significant.


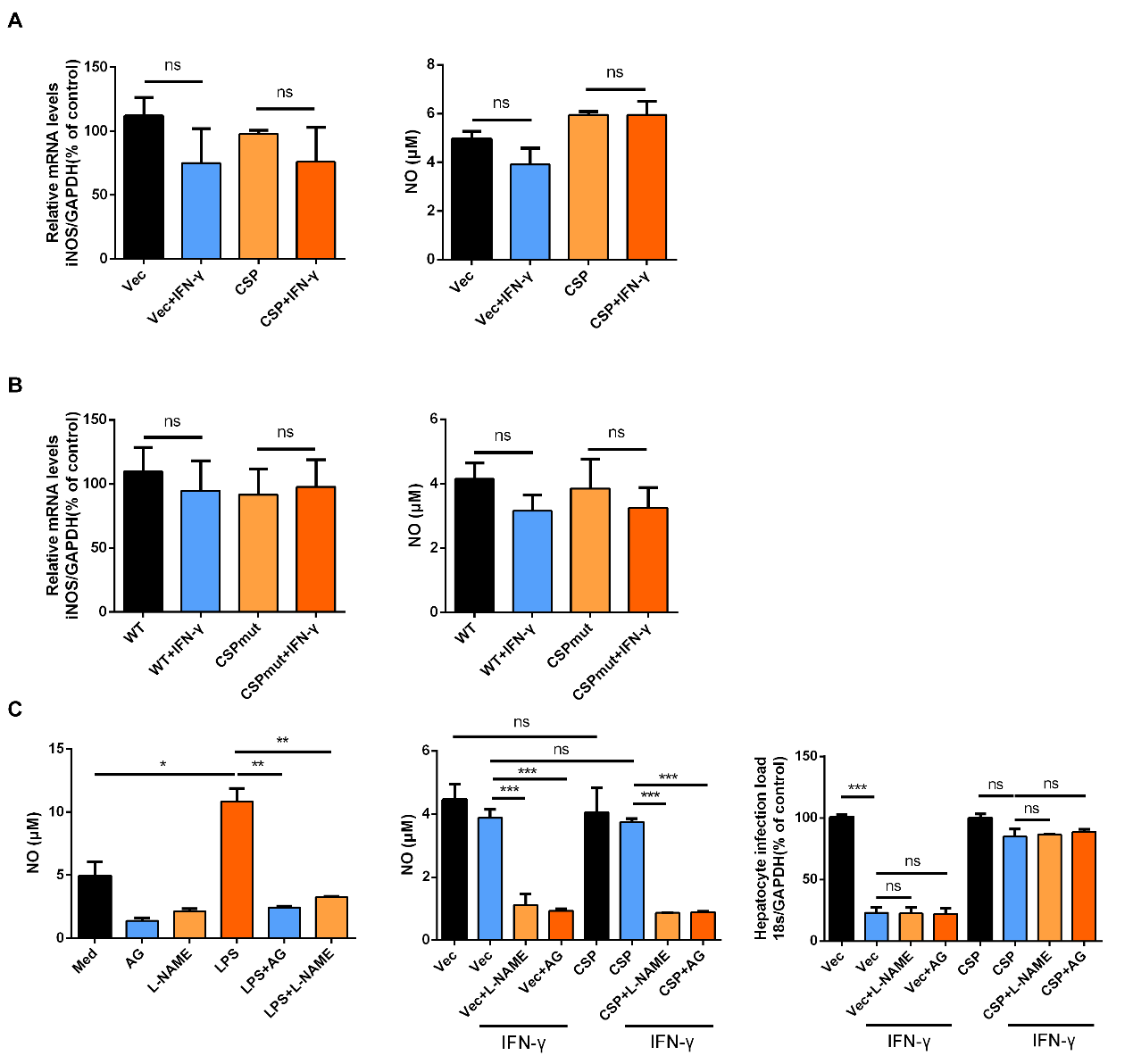


**Figure S4 NO is not involved in the suppression of the IFN-γ-mediated killing of EEFs by CSP.**

(**A**) 1.2 × 10^5^ HepG2 cells were transfected with pcDNA3.1 (Vec) or pcDNA3.1-CSP plasmid and treated with or without 1U/mL IFN-γ, and then infected with 4 × 10^4^ *P.b* ANKA sporozoites for 24 h. The mRNA level of iNOS (*left*) and the concentration of NO in the supernatants (*right*) were measured by real-time qPCR and the Griess reaction assay, respectively. (**B**) 1.2 × 10^5^ HepG2 cells were pre-treated with or without 1U/mL IFN-γ and incubated with 4 × 10^4^ CSP_wt_ or CSP_mut_ sporozoites for 24 h. The mRNA level of iNOS (*left*) and concentration of NO (*right*) produced by CSP_wt_ or CSP_mut_ parasite-infected hepatocytes were measured as described as above. (**C**) RAW264.7 cells were pretreated with or without the iNOS inhibitor Aminoguanidine (AG) or L-NAME and then stimulated with LPS for 4 h. The concentration of NO in the supernatants of LPS-treated macrophages was measured (*left*). 1.2 × 10^5^ HepG2 cells were transfected with pcDNA3.1 (Vec) or pcDNA3.1-CSP plasmid and treated with or without the iNOS inhibitor AG or L-NAME and 1U/mL IFN-γ followed by infection with 4 × 10^4^ *P.b* ANKA sporozoites. The concentration of NO (*middle*) and the parasite load (*right*) were determined by 24 h and 46 h after infection, respectively. Each experiment was repeated for three times. Data are represented as mean ± SD, and analyzed by One-way ANOVA; Med, medium; Vec, vector; ns, not significant; **P* < 0.05, ***P* < 0.01, ****P* < 0.001.


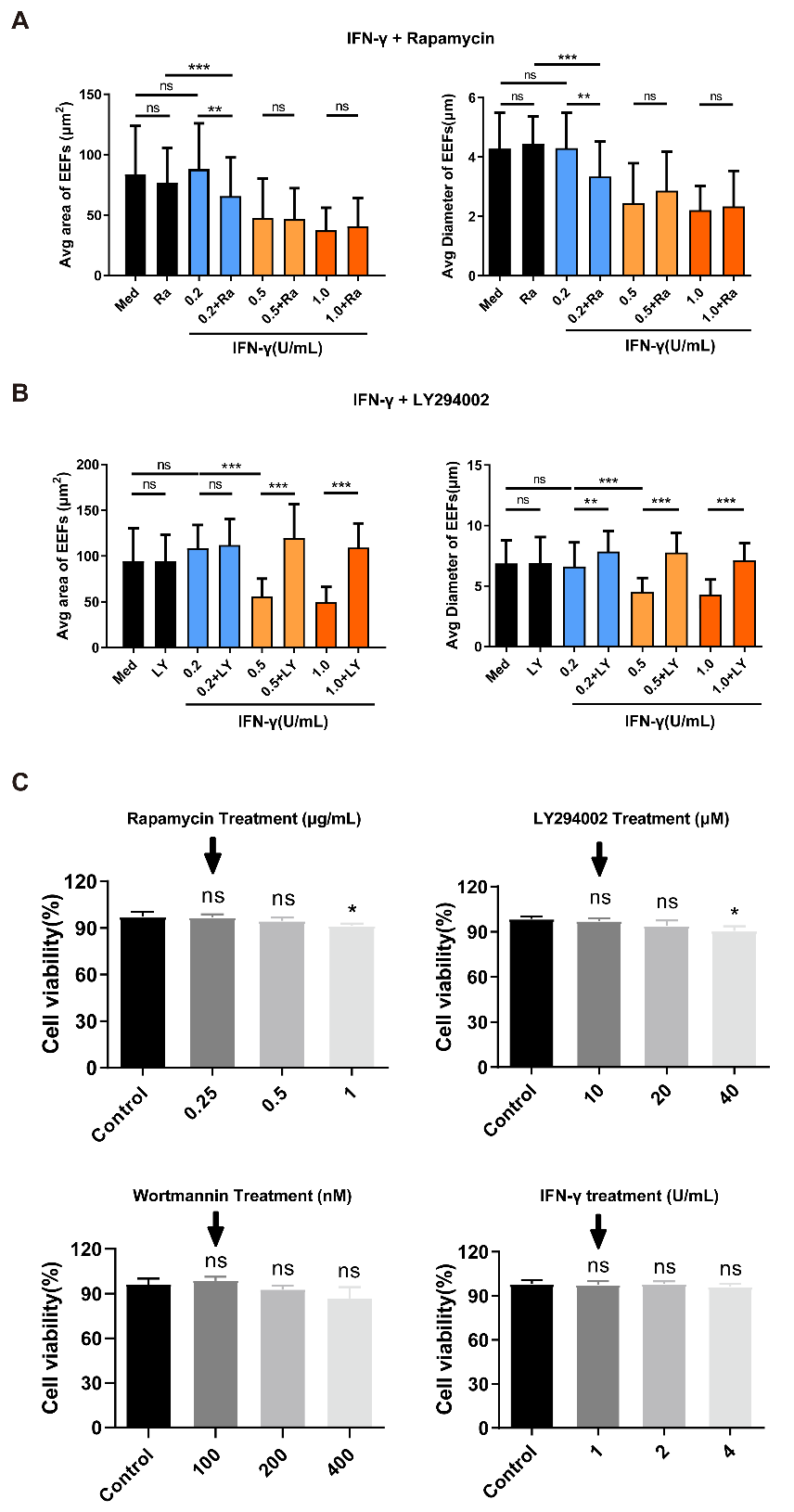


**Figure S5** **The effect of the autophagy modulators on the IFN-γ-suppression of EFFs development.**

(**A**) 1.2 × 10^5^ HepG2 cells were pre-treated with or without the autophagy inducer rapamycin (Rapa) and IFN-γ at the indicated concentrations and then incubated with 4 × 10^4^ sporozoites. The size (*left*) and the diameter (*right*) of EEFs at 24 h post-infection was compared, n=22-49, magnification, 630×. (**B**) 1.2 × 10^5^ HepG2 cells were pre-treated with or without the autophagy inhibitor LY294002 (LY) and IFN-γ at the indicated concentrations and then incubated with 4 × 10^4^ sporozoites. The size (*left*) and diameter (*right*) of EEFs at 24 h post-infection was compared, n=36-55, magnification, 630×. (**C**) CCK-8 analysis results showed that chemical agents used as mentioned above in these experiments have no influences on the cell viability of HepG2 cells (Black arrow indicates the concentration of chemical agents used in our study). Two-three independent experiments were performed for each experiment. Data are represented as mean ± SEM, and analyzed by One-way ANOVA; ns, not significant; **P <* 0.05; ***P<*0.01; ****P<*0.001.

**
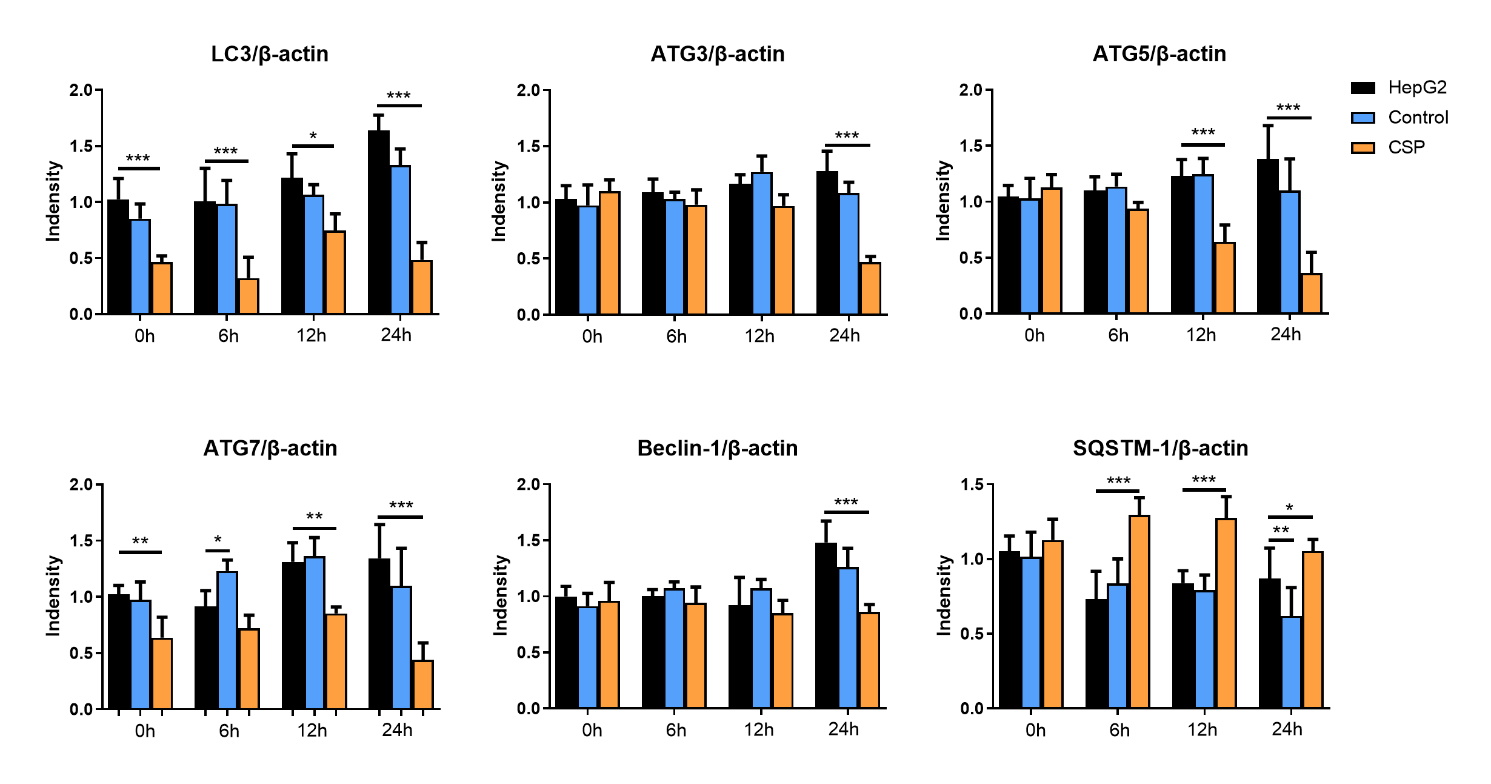
**

**Figure S6** **Quantitative analysis of intensity of autophagy-related proteins.**

The bar graph presents the relative expression of LC3, ATG3, ATG5, ATG7, Beclin-1, and SQSTM-1 calculated from densitometric measurements of three independent western blot analyses. The intensity of ATG proteins was normalized to 0h in each group (Related to **Figure 3C**). **P <* 0.05; ***P<*0.01; ****P<*0.001.


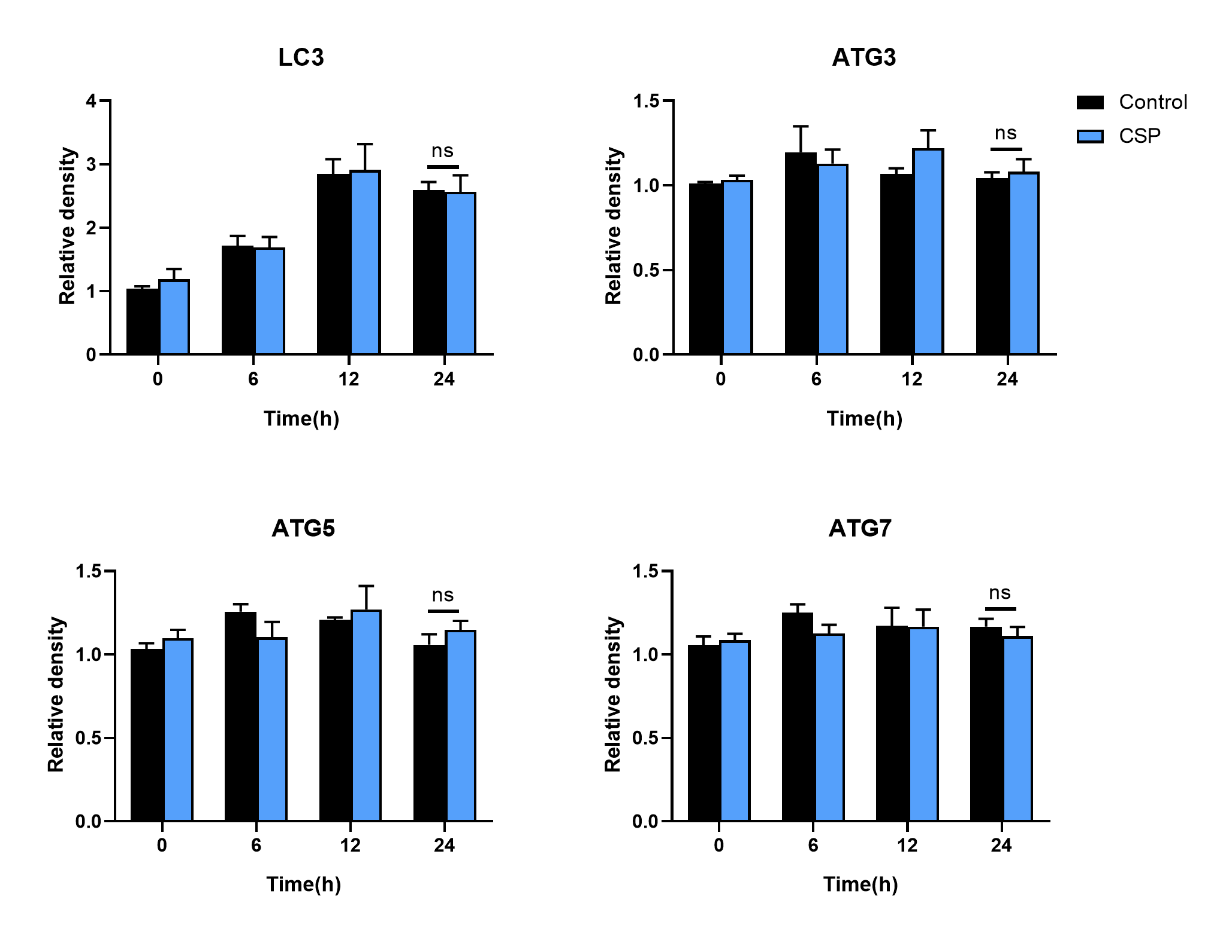


**Figure S7** **Quantitative analysis of intensity of autophagy-related proteins.**

The bar graph presents the relative expression of LC3, ATG3, ATG5, ATG7 calculated from densitometric measurements of three independent western blot analyses. The intensity of ATG proteins was normalized to 0h in each group (Related to **Figure 4D**). ns, not significant.

**
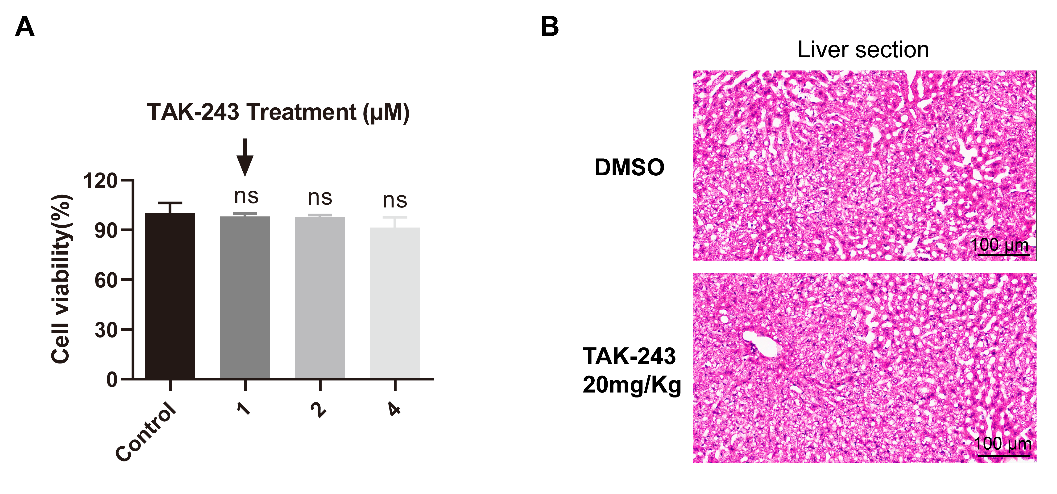
**

**Figure S8 Cytotoxic effect of TAK-243 at the indicated concentrations.**

(**A**) The effects of 1, 2, and 4 μM of TAK-243 on the cell viability of HepG2 cells were detected by CCK-8 analysis (Black arrow indicates the concentration of TAK-243 used in our study). Data are represented as mean ± SD, and analyzed by One-way ANOVA; ns, not significant. (**B**) Mouse was injected with or without 20mg/kg TAK-243. After 46h, livers were dissected, embedded in paraffin sectioned, and dewaxed. The sections were stained with hematoxylin and eosin. The liver structure was observed and photographed. TAK-243 was dissolved in DMSO and then diluted with PBS. Scale bar = 100μm.


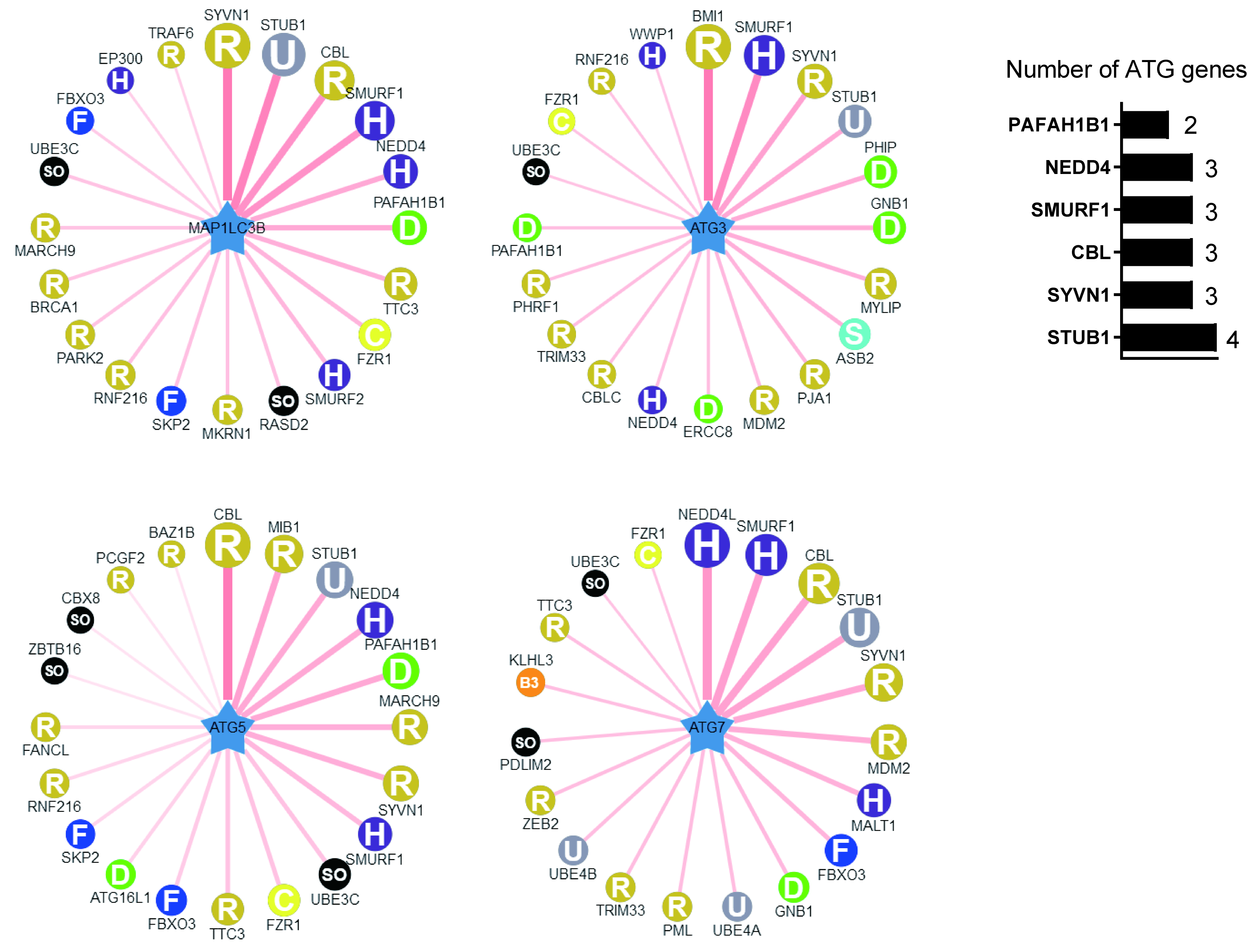


**Figure S9 The prediction of E3 ubiquitin ligases possibly involved in the ubiquitination of ATGs.**

E3 ubiquitin ligases possibly involved in the ubiquitination of ATGs were predicted online (http://ubibrowser.ncpsb.org/ubibrowser/) (*left*), and E3 ubiquitin ligases predicted to regulate at least two ATGs (LC3, ATG3, ATG5, and ATG7) are listed in the right column. Numbers indicate the number of ATG proteins regulated by the six predicted E3 ubiquitin ligases, including STUB1, SYVN1, CBL, SMURF1, PAFAH1B1 and NEDD4, respectively. (*right*).


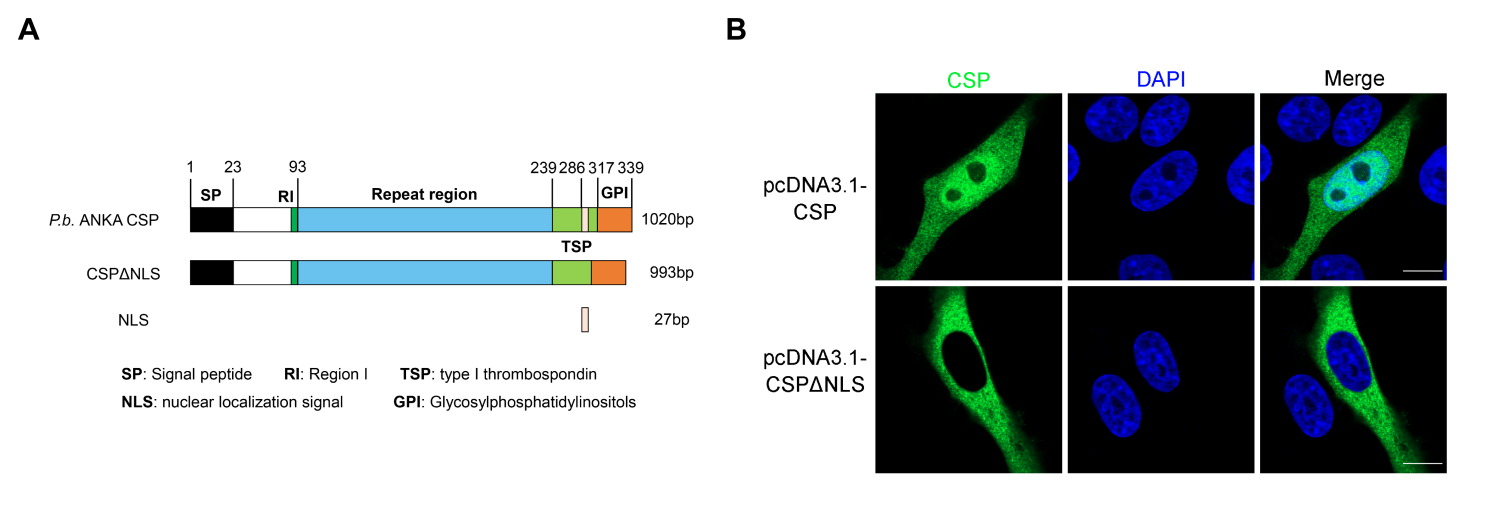


**Figure S10 The illustration of CSP and its truncated forms and their distribution after transfection into HepG2.**

(**A**) Schematic view of *P.b* ANKA CSP (Uniprot Entry: P06915), CSPΔNLS and NLS used in this study. **SP**, signal peptide; **RI**: Region I; **TSP**: type I thrombospondin; **NLS**, nuclear localization signal. **GPI**, glycosylphosphatidyl inositol attachment site. (**B**) HepG2 cells were transfected with pcDNA3.1-CSP-HA or pcDNA3.1-CSPΔNLS-HA plasmids, 24 h later, the distribution of CSP protein was labeled by rabbit anti-HA antibody (1:500) and IFKine Green AffiniPure donkey anti-rabbit IgG (H+L) (1:100). Scale bar = 10 μm.


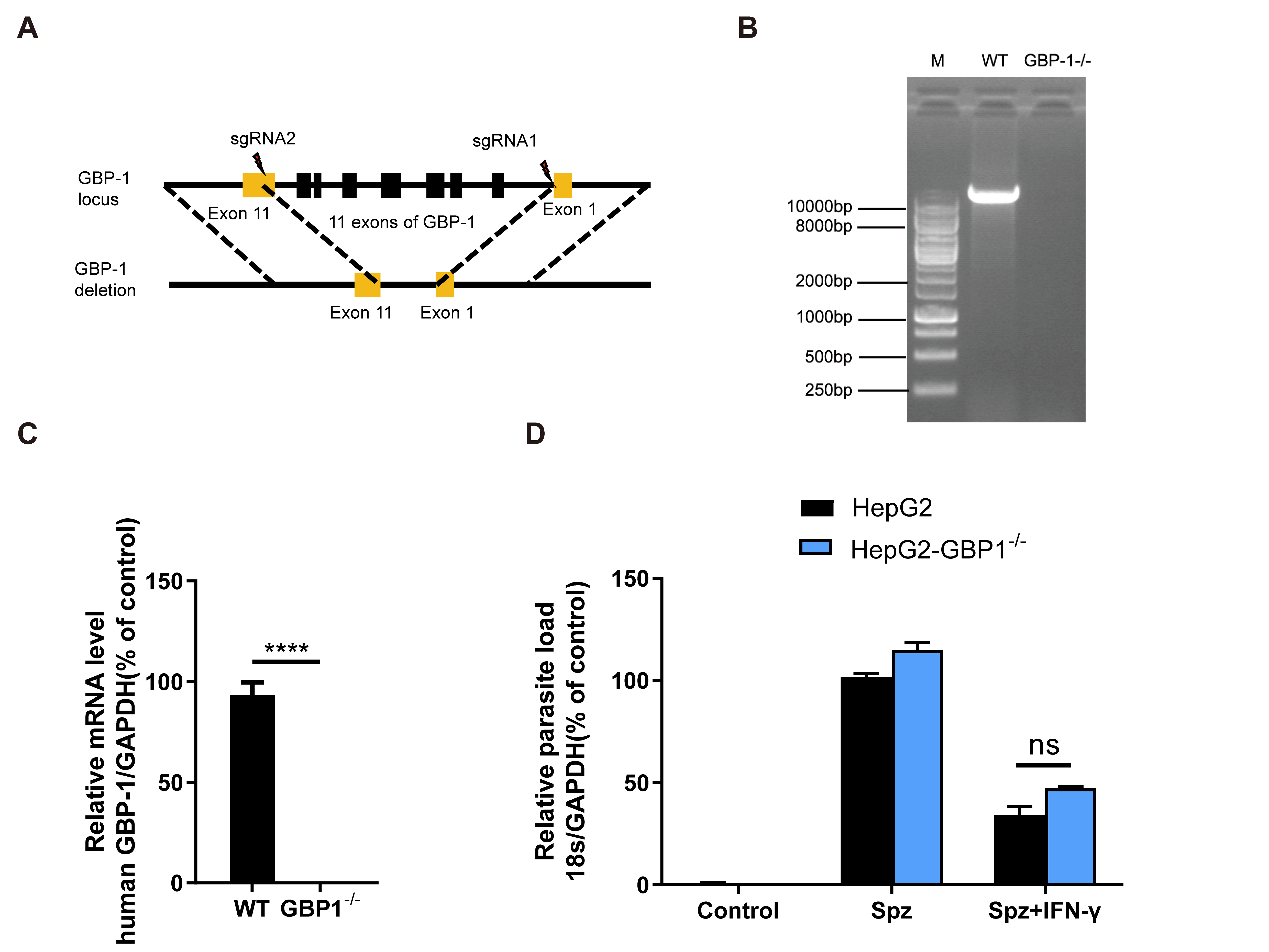


**Figure S11 GBP1 is dispensable for the IFN-γ-mediated killing of EEFs.**

(**A**) Schematic representation of the disruption of *GBP1* gene by CRISPR-Cas9. (**B**) The identification of *GBP1*^-/-^ HepG2 cells by PCR with specific primers binding to the left side of the sgRNA2 and right side of sgRNA1 targeting site in *GBP-1* exons (PCR fragment of wide type is 11,448bp). (**C**) The relative mRNA level of *GBP1* to *GAPDH* was detected in WT and *GBP1*^-/-^ HepG2 cells by real-time PCR. (**D**) 1.2 × 10^5^ WT and *GBP1*^-/-^ HepG2 cells were infected with 4 × 10^4^ *P.b* ANKA sporozoites for 46 h, and then treated with 1U/mL IFN-γ. The parasite load in WT and *GBP1*^-/-^ HepG2 cells was measured and compared as described before. Two independent experiments have been performed. Data are represented as mean ± SD and analyzed by a student's t-test or Mann-Whitney U test; spz, sporozoites; ns, not significant. *****P* < 0.0001.

## Supplementary Tables

| **Table S1. Numbers of mutations detected at the potential off-target cleavage sites in the *Plasmodium* *berghei* ANKA genome.** | | | | | |
| --- | --- | --- | --- | --- | --- |
| sgRNA |  | Sequence | No. of mismatches | Chromosome location | Mutation |
| CSP targeting sgRNA |  | GTAGCTCGTTTAAGTTCCTT**TGG** | None |  |  |
|  | 1 | GTAGATGTTTTAATTTCATT**TGG** | 5 | 7: 755321-755343(+) | ND |
|  | 2 | GTAATTCGTATAAATTCGTT**TGG** | 5 | 14: 1448473-1448495(+) | ND |
|  | 3 | GTAGATATTTTTTGTTCCAT**TGG** | 6 | 6: 283460-283481(+) | ND |
|  | 4 | GTATGTCATTTGGTTTCCTT**TGG** | 6 | 12: 1205398-1205420(-) | ND |
|  | 5 | TTTTCTCTTTTAATTTCCAT**TGG** | 6 | 13: 669526-669547(+) | ND |
|  | 6 | GTATATCGTTTCGTTTCCCT**TGG** | 6 | 13: 1991271-1991293(-) | ND |
|  | 7 | TTATCTCGTTGCCTTTCCTT**TGG** | 6 | 14: 2441124-2441146(-) | ND |
| Note: the underlined bases are mismatches with the CSP targeting sgRNA. ND, not detected. | | | | | |

| **Table S2. Scoring of E3 ubiquitin ligases possibly involved in the ubiquitination of each ATGs.** | | | | | | | | | |
| --- | --- | --- | --- | --- | --- | --- | --- | --- | --- |
| **LC3** | | | | | | | | | |
| E3 | E3GENE | SUB | SUBGENE | HOMO | PFAM | GO | NET | MOTIF | SCORE |
| Q86TM6 | SYVN1 | Q9GZQ8 | MAP1LC3B | 1 | 1 | 1.25 | 1 | 6.61 | 0.714 |
| Q9UNE7 | STUB1 | Q9GZQ8 | MAP1LC3B | 1 | 1 | 3.77 | 1.84 | 1 | 0.699 |
| P22681 | CBL | Q9GZQ8 | MAP1LC3B | 1 | 1 | 3.77 | 1.44 | 1 | 0.676 |
| Q9HCE7 | SMURF1 | Q9GZQ8 | MAP1LC3B | 1 | 1 | 2.88 | 2.3 | 1.06 | 0.675 |
| P43034 | PAFAH1B1 | Q9GZQ8 | MAP1LC3B | 1 | 1 | 3.77 | 1 | 1 | 0.64 |
| P46934 | NEDD4 | Q9GZQ8 | MAP1LC3B | 1 | 1 | 3.77 | 1 | 1 | 0.64 |
| P53804 | TTC3 | Q9GZQ8 | MAP1LC3B | 1 | 1 | 1.25 | 1.29 | 2.12 | 0.63 |
| Q9UM11 | FZR1 | Q9GZQ8 | MAP1LC3B | 1 | 1 | 1.51 | 1 | 2.12 | 0.624 |
| Q9HAU4 | SMURF2 | Q9GZQ8 | MAP1LC3B | 1 | 1 | 1.78 | 1.77 | 1 | 0.622 |
| Q96D21 | RASD2 | Q9GZQ8 | MAP1LC3B | 1 | 1 | 1.51 | 1.84 | 1 | 0.609 |
| Q9NWF9 | RNF216 | Q9GZQ8 | MAP1LC3B | 1 | 1 | 1 | 1.29 | 2.12 | 0.608 |
| Q9UHC7 | MKRN1 | Q9GZQ8 | MAP1LC3B | 1 | 1 | 1 | 1.29 | 2.12 | 0.608 |
| Q13309 | SKP2 | Q9GZQ8 | MAP1LC3B | 1 | 1 | 1 | 1.29 | 2.12 | 0.608 |
| P38398 | BRCA1 | Q9GZQ8 | MAP1LC3B | 1 | 1 | 1.51 | 1.77 | 1 | 0.605 |
| O60260 | PARK2 | Q9GZQ8 | MAP1LC3B | 1 | 1 | 1.51 | 2.3 | 1 | 0.605 |
| Q86YJ5 | 9-Mar | Q9GZQ8 | MAP1LC3B | 1 | 1 | 1.25 | 1 | 2.12 | 0.604 |
| Q15386 | UBE3C | Q9GZQ8 | MAP1LC3B | 1 | 1 | 1.25 | 1 | 2.12 | 0.604 |
| Q9UK99 | FBXO3 | Q9GZQ8 | MAP1LC3B | 1 | 1 | 1.13 | 1 | 2.12 | 0.594 |
| Q09472 | EP300 | Q9GZQ8 | MAP1LC3B | 1 | 1 | 1.25 | 1.84 | 1 | 0.589 |
| Q9Y4K3 | TRAF6 | Q9GZQ8 | MAP1LC3B | 1 | 1 | 1.25 | 2.39 | 1 | 0.589 |
| **ATG3** | | | | | | | | | |
| E3 | E3GENE | SUB | SUBGENE | HOMO | PFAM | GO | NET | MOTIF | SCORE |
| P35226 | BMI1 | Q9NT62 | ATG3 | 1 | 1 | 1.25 | 1 | 6.61 | 0.714 |
| Q9HCE7 | SMURF1 | Q9NT62 | ATG3 | 1 | 1 | 4.05 | 1.44 | 1.06 | 0.688 |
| Q86TM6 | SYVN1 | Q9NT62 | ATG3 | 1 | 1 | 1.25 | 1 | 3.41 | 0.652 |
| Q9UNE7 | STUB1 | Q9NT62 | ATG3 | 1 | 1 | 3.77 | 1 | 1 | 0.64 |
| Q8WWQ0 | PHIP | Q9NT62 | ATG3 | 1 | 1 | 2.88 | 1.29 | 1 | 0.639 |
| P62873 | GNB1 | Q9NT62 | ATG3 | 1 | 1 | 2.88 | 1.29 | 1 | 0.639 |
| Q8WY64 | MYLIP | Q9NT62 | ATG3 | 1 | 1 | 1.25 | 1 | 2.8 | 0.633 |
| Q96Q27 | ASB2 | Q9NT62 | ATG3 | 1 | 1 | 1 | 1 | 3.41 | 0.63 |
| Q8NG27 | PJA1 | Q9NT62 | ATG3 | 1 | 1 | 1.13 | 1 | 2.8 | 0.623 |
| Q00987 | MDM2 | Q9NT62 | ATG3 | 1 | 1 | 2.88 | 1 | 1.06 | 0.619 |
| Q13216 | ERCC8 | Q9NT62 | ATG3 | 1 | 1 | 2.88 | 1 | 1 | 0.613 |
| Q9P1Y6 | PHRF1 | Q9NT62 | ATG3 | 1 | 1 | 2.88 | 1 | 1 | 0.613 |
| Q9ULV8 | CBLC | Q9NT62 | ATG3 | 1 | 1 | 2.88 | 1 | 1 | 0.613 |
| P43034 | PAFAH1B1 | Q9NT62 | ATG3 | 1 | 1 | 2.88 | 1 | 1 | 0.613 |
| P46934 | NEDD4 | Q9NT62 | ATG3 | 1 | 1 | 2.88 | 1 | 1 | 0.613 |
| Q9UPN9 | TRIM33 | Q9NT62 | ATG3 | 1 | 1 | 2.88 | 1 | 1 | 0.613 |
| Q9H0M0 | WWP1 | Q9NT62 | ATG3 | 1 | 1 | 1.25 | 1 | 2.12 | 0.604 |
| Q9NWF9 | RNF216 | Q9NT62 | ATG3 | 1 | 1 | 1.25 | 1 | 2.12 | 0.604 |
| Q5XUX0 | FBXO31 | Q9NT62 | ATG3 | 1 | 1 | 1.25 | 1 | 2.12 | 0.604 |
| Q15386 | UBE3C | Q9NT62 | ATG3 | 1 | 1 | 1.25 | 1 | 2.12 | 0.604 |
| Q9UM11 | FZR1 | Q9NT62 | ATG3 | 1 | 1 | 1.25 | 1 | 2.12 | 0.604 |
| Q14258 | TRIM25 | Q9NT62 | ATG3 | 1 | 1 | 1.25 | 1 | 2.12 | 0.604 |
| Q9C040 | TRIM2 | Q9NT62 | ATG3 | 1 | 1 | 1.25 | 1 | 2.12 | 0.604 |
| **ATG5** | | | | | | | | | |
| E3 | E3GENE | SUB | SUBGENE | HOMO | PFAM | GO | NET | MOTIF | SCORE |
| P22681 | CBL | Q9H1Y0 | ATG5 | 1 | 1 | 3.77 | 1 | 2.12 | 0.711 |
| Q86YT6 | MIB1 | Q9H1Y0 | ATG5 | 1 | 1 | 1.51 | 1 | 2.8 | 0.652 |
| Q9UNE7 | STUB1 | Q9H1Y0 | ATG5 | 1 | 1 | 3.77 | 1 | 1 | 0.64 |
| P46934 | NEDD4 | Q9H1Y0 | ATG5 | 1 | 1 | 3.77 | 1 | 1 | 0.64 |
| P43034 | PAFAH1B1 | Q9H1Y0 | ATG5 | 1 | 1 | 3.77 | 1 | 1 | 0.64 |
| Q86YJ5 | MARCH9 | Q9H1Y0 | ATG5 | 1 | 1 | 1.25 | 1 | 2.8 | 0.633 |
| Q86TM6 | SYVN1 | Q9H1Y0 | ATG5 | 1 | 1 | 1.25 | 1 | 2.8 | 0.633 |
| Q9HCE7 | SMURF1 | Q9H1Y0 | ATG5 | 1 | 1 | 2.88 | 1 | 1.06 | 0.619 |
| Q9UM11 | FZR1 | Q9H1Y0 | ATG5 | 1 | 1 | 1.25 | 1 | 2.12 | 0.604 |
| P53804 | TTC3 | Q9H1Y0 | ATG5 | 1 | 1 | 1.25 | 1 | 2.12 | 0.604 |
| Q15386 | UBE3C | Q9H1Y0 | ATG5 | 1 | 1 | 1.25 | 1 | 2.12 | 0.604 |
| Q9UK99 | FBXO3 | Q9H1Y0 | ATG5 | 1 | 1 | 1.13 | 1 | 2.12 | 0.594 |
| Q676U5 | ATG16L1 | Q9H1Y0 | ATG5 | 1 | 1 | 1.25 | 2.09 | 1 | 0.585 |
| Q9NWF9 | RNF216 | Q9H1Y0 | ATG5 | 1 | 1 | 1 | 1 | 2.12 | 0.581 |
| Q9NW38 | FANCL | Q9H1Y0 | ATG5 | 1 | 1 | 1 | 1 | 2.12 | 0.581 |
| Q13309 | SKP2 | Q9H1Y0 | ATG5 | 1 | 1 | 1 | 1 | 2.12 | 0.581 |
| Q05516 | ZBTB16 | Q9H1Y0 | ATG5 | 1 | 1 | 1.25 | 1.44 | 1 | 0.563 |
| Q9UIG0 | BAZ1B | Q9H1Y0 | ATG5 | 1 | 1 | 1.78 | 1 | 1 | 0.562 |
| P35227 | PCGF2 | Q9H1Y0 | ATG5 | 1 | 1 | 1.78 | 1 | 1 | 0.562 |
| Q9HC52 | CBX8 | Q9H1Y0 | ATG5 | 1 | 1 | 1.78 | 1 | 1 | 0.562 |
| **ATG7** | | | | | | | | | |
| E3 | E3GENE | SUB | SUBGENE | HOMO | PFAM | GO | NET | MOTIF | SCORE |
| Q96PU5 | NEDD4L | O95352 | ATG7 | 1 | 1 | 2.33 | 1 | 2.12 | 0.667 |
| Q9HCE7 | SMURF1 | O95352 | ATG7 | 1 | 1 | 2.88 | 1.44 | 1.06 | 0.655 |
| P22681 | CBL | O95352 | ATG7 | 1 | 1 | 1.51 | 1 | 2.8 | 0.652 |
| Q9UNE7 | STUB1 | O95352 | ATG7 | 1 | 1 | 4.05 | 1 | 1 | 0.647 |
| Q86TM6 | SYVN1 | O95352 | ATG7 | 1 | 1 | 1.13 | 1 | 3.41 | 0.642 |
| Q00987 | MDM2 | O95352 | ATG7 | 1 | 1 | 1.27 | 1 | 2.8 | 0.634 |
| Q9UDY8 | MALT1 | O95352 | ATG7 | 1 | 1 | 2.33 | 1.44 | 1 | 0.628 |
| Q9UK99 | FBXO3 | O95352 | ATG7 | 1 | 1 | 1.13 | 1 | 2.8 | 0.623 |
| P62873 | GNB1 | O95352 | ATG7 | 1 | 1 | 2.33 | 1.29 | 1 | 0.617 |
| O60315 | ZEB2 | O95352 | ATG7 | 1 | 1 | 2.88 | 1 | 1 | 0.613 |
| Q14139 | UBE4A | O95352 | ATG7 | 1 | 1 | 2.88 | 1 | 1 | 0.613 |
| P29590 | PML | O95352 | ATG7 | 1 | 1 | 2.88 | 1 | 1 | 0.613 |
| O95155 | UBE4B | O95352 | ATG7 | 1 | 1 | 2.88 | 1 | 1 | 0.613 |
| Q9UPN9 | TRIM33 | O95352 | ATG7 | 1 | 1 | 2.88 | 1 | 1 | 0.613 |
| Q96JY6 | PDLIM2 | O95352 | ATG7 | 1 | 1 | 1 | 1 | 2.8 | 0.61 |
| P53804 | TTC3 | O95352 | ATG7 | 1 | 1 | 1 | 1 | 2.8 | 0.61 |
| Q9UH77 | KLHL3 | O95352 | ATG7 | 1 | 1 | 1 | 1 | 2.8 | 0.61 |
| Q15386 | UBE3C | O95352 | ATG7 | 1 | 1 | 1.25 | 1 | 2.12 | 0.604 |
| Q9UM11 | FZR1 | O95352 | ATG7 | 1 | 1 | 1.25 | 1 | 2.12 | 0.604 |

# 2 Supplementary Materials and Methods

**2.1 Construction of CSP pexel I-II mutation parasite**

The *P. b ANKA* CSP_mut_ parasite was constructed by replacing the WT *CSP* sequence with a pexel I-II mutant *CSP* using CRISPR-Cas9. Single-guide RNA (5-ATAGCTCGTTTAAGTTCCTT-3) specifically targeting the *P.b.* ANKA *CSP* gene (Gene ID in PlasmoDB database PBANKA_0403200) was inserted downstream of the *Plasmodium* U6 promoter in the pYC plasmid (a gift from Dr. Jin Yuan, Xia’men University, China). The homologous recombinant fragment for the CSP pexel mutation containing the 5 untranslated region (593 bp) and coding sequencing with mutant pexel-I/II of the *CSP* locus (1023 bp) was constructed by overlapping PCR and inserted into the multiple cloning sites of the pYC plasmid. *P.b* ANKA CSP pexel domain I-II was mutated as previously reported.^1^ The amino acid sequence of pexel domain I, RNLNE, was changed to ANANA, while the sequence of pexel domain II, RLLAD, was changed to ALAAA. The resulting recombinant pYC-CSP_mut_ plasmid was amplified and purified using an Endo-Free Plasmid Midi Kit (Omega, Norcross, GA, USA). Plasmid electro-transfection was performed as previously reported.^2^ In brief, Kunming mice were infected with *P. b* ANKA via intraperitoneal injection, and the erythrocytic-stage parasites were collected from the mice with parasitemia of 5%–15%. The parasites were immediately added to a 250-mL conical flask containing 100 ml RPMI-1640 culture medium (HyClone, Logan, UT, USA) and 25 mL fetal bovine serum (FBS; Gibco, Detroit, MI, USA), and then a gas mixture of 5% CO_2_, 5% O_2_, and 90% N_2_ was flushed into the conical flask using a 0.22-μm filter (Millipore, Billerica, MA, USA). The conical flask was incubated at 37°C on a swing bed with shaking at a speed that was high enough to keep the cells in suspension overnight. Subsequently, the schizonts were separated and collected by density gradient centrifugation with 72% Percoll (GE Healthcare, Amersham, Buckinghamshire, UK). The Human T Cell Nucleofector^®^ Kit (Lonza, Basel, Switzerland) was used for electro-transfection. The schizonts were resuspended in 110 μL DNA solution containing 100 μL nucleofector solution and 10 μL ddH_2_O containing 10–20 μg recombinant pYC-CSP_mut_ plasmid and transfected using program U33. Fifty microliters of complete culture medium were added immediately and 150 μL of the complete transfection solution was injected into a tail vein of a Kunming mouse.

Once parasites appeared in the blood, the mice were fed 8 μg/mL pyrimethamine (Sigma-Aldrich, St Louis, MO, USA) diluted in water to screen for mutant parasites. The pyrimethamine-resistant parasites were collected and cloned by intravenously injecting each mouse with 100 μL of a phosphate-buffered saline-diluted parasite solution containing ~1.0 infected iRBC. Nine to 12 days later, the blood of the mice with cloned parasites was collected and genomic DNA was extracted. The CSP pexel I-II mutation of the parasite clone was identified by PCR from the genomic DNA, followed by verification with DNA sequencing.

**2.2 Griess reaction analysis**

The supernatant of the cell culture was collected 24 h after infection with sporozoites, transfected with pcDNA3.1 or pcDNA3.1-CSP, and treated with or without IFN-γ. For Griess analysis (Beyotime), the NaNO_2_ standard was diluted in DMEM containing 10% FBS to a final concentration of 0, 1, 2, 5, 10, 20, 40, 60, and 100 µM. Fifty microliters of different concentrations of NaNO_2_ standards and the supernatant were added to a 96-well plate, and then 50 μL of Griess Reagent I and II (Beyotime) were added sequentially at room temperature. After slight vibration, the concentration of NO was measured at 540 nm using an iMark^TM^ Microplate Absorbance Reader (Bio-Rad). RAW264.7 cells stimulated with 100 ng/mL LPS (Sigma-Aldrich) were used as positive controls.

References

1. Matthias Marti, Robert T Good, Melanie Rug, Ellen Knuepfer, Alan F Cowman. Targeting malaria virulence and remodeling proteins to the host erythrocyte. Science. 2004, 10;306(5703):1930-3.

2. Janse CJ, Ramesar J, Waters AP. High-efficiency transfection and drug selection of genetically transformed blood stages of the rodent malaria parasite Plasmodium berghei. Nat Protoc 2006, 1(1): 346-356.
